# Rationally designed nanotrap structures for efficient separation of rare earth elements over a single step

**DOI:** 10.1038/s41467-024-45810-1

**Published:** 2024-02-20

**Authors:** Qing-Hua Hu, An-Min Song, Xin Gao, Yu-Zhen Shi, Wei Jiang, Ru-Ping Liang, Jian-Ding Qiu

**Affiliations:** 1 State Key Laboratory of Nuclear Resources and Environment, East China University of Technology, Nanchang, China; 2 School of Chemistry and Chemical Engineering, Nanchang University, Nanchang, China; 3 School of Chemistry and Chemical Engineering, Jinggangshan University, Ji’an, China

**Keywords:** Polymers, Chemical bonding, Organic molecules in materials science

## Abstract

Extracting rare earth elements (REEs) from wastewater is essential for the growth and an eco-friendly sustainable economy. However, it is a daunting challenge to separate individual rare earth elements by their subtle differences. To overcome this difficulty, we report a unique REE nanotrap that features dense uncoordinated carboxyl groups and triazole N atoms in a two-fold interpenetrated metal-organic framework (named NCU-1). Notably, the synergistic effect of suitable pore sizes and REE nanotraps in NCU-1 is highly responsive to the size variation of rare-earth ions and shows high selectivity toward light REE. As a proof of concept, Pr/Lu and Nd/Er are used as binary models, which give a high separation factor of *SF*_Pr/Lu_ = 796 and *SF*_Nd/Er_ = 273, demonstrating highly efficient separation over a single step. This ability achieves efficient and selective extraction and separation of REEs from mine tailings, establishing this platform as an important advance for sustainable obtaining high-purity REEs.

## Abstract (web summary)

Extracting rare earth elements (REEs) from wastewater is essential for the growth of an eco-friendly sustainable economy but separating individual rare earth elements remains challenging. Here, the authors report a REE nanotrap that features dense uncoordinated carboxyl groups and triazole N atoms in a two-fold interpenetrated metalorganic framework which is highly responsive to the size variation of rareearth ions.

## Introduction

Rare-earth elements (REEs) are used in a wealth of high-tech applications, including permanent magnets, lasers, phosphors, catalytic converters, and batteries^1–4^, owing to their 4 f peculiar electronic structure. To meet the REEs demands of the emerging clean energy technology market, recovery and separation of REEs from wastes is an eco-friendly sustainable strategy. However, achieving their selective separation and purification still faces a great challenge due to the large excess of interfering ions, low REE concentrations, and their highly similar chemical properties^5,6^.

Various approaches have been utilized to separate REEs. Fractional crystallization, as one of the earliest separation methods, which has low separation efficiency, owning to its driving force originates primarily from the ionic radii differences^7,8^. Currently, solvent extraction^9–11^, and liquid membrane separation methods^12,13^ have been utilized for the industrial separation REEs. A high separation factor can be achieved by hundreds of stages while it often faces problems such as low efficiency and generating massive of toxic secondary organic liquid waste^14^. Given this, several strategies have emerged to enhance the separation efficiency of REEs. For example, Schelter et al. based on the differences in the equilibrium constants of early and late metals complex with a tripodal nitroxide ligand to enhance the separation factor of Nd/Dy^15^. Bu et al. utilized the size-selective crystallization of rare-earth ions method to amplify the difference between early and late lanthanides^16,17^. Wang et al. controlled the reaction kinetics to separate Nd/Sm and Nd/Dy during the crystallization of borates^3^. However, these methods share many shortages of high temperature, long incubation times, and low efficiency, which hinder scale-up production and practical application^3,16^. Compared with the aforementioned methods, an adsorption method could overcome the above limits due to its easy operation, intrinsic economic feasibility, and little secondary pollution^18^. Traditional adsorbents of activated carbon^19^, metal oxides^20^, and composites^21^ have been developed for REEs extractive and separation, but they usually suffer from low selectivity and capacity because of their low adsorb sites or unsuitable pore sizes^22^. Therefore, integrating appropriate apertures and high-density active sites within one adsorbent is the key to enhancing the separation efficiency of REEs. Towards this end, metal-organic frameworks (MOFs) provided new opportunities for REEs separation owing to their tunable pore sizes and diverse functionalities at the molecular scale^23–26^. Given the fact that light REE ions exhibit a slightly larger size than the heavy REE ions and interfering ions in mine tailings^27,28^, we reasoned that it is essential to design a suitable aperture or form multiple interactions to enhance the interaction between the light/heavy REE ions and the host framework for efficient REE ions separation. Although these promising properties of MOFs for separation, only a few cases of the separation of REEs by MOFs have been reported. Recently, two hydroxyl-rich 0.8 nm hexagonal channel MOFs were reported for selective separation of lanthanides by solid-phase extraction, where the separation factor was only less than 2, ascribing to the weak affinity between REEs with hydroxyl groups^29^. Qiu et al. synthesized a Zn-BTC MOF/NG using a solid-phase in situ synthesis strategy, exhibiting excellent separation selectivity of REEs, which attributed to the synergistic effects of the three-dimensional pore of MOF and two-dimensional pore of NG^30^. Ahn et al. prepared a chromium-based MOF (Cr-MIL-101), and its derivatives with different organic functional groups for the adsorption of several REE ions. It demonstrated that the carboxylate (−COO − ), phosphonic (−PO_3_^2−^), and nitrogen-containing groups have a strong coordinative affinity toward REE ions^31^. However, the reported materials have large pore sizes ranging from 1.2 to 2.2 nm, which were ineffective for the separation of different REEs^31^. It was reported that the crown ether (18-C-6) or macrocyclic molecules with about 3.2 Å cavity diameters show good selectivity for the large Ln^3+^ ions, due to the better matching of the crown ether cavity size with Ln^3+^ ions diameter^32–34^. We hypothesized that if the REE nanotrap is immobilized on the suitable pore size MOFs, it will not only provide strong interactions to capture REE ions but also obtain pure individual REE ions.

**Fig. 1:**
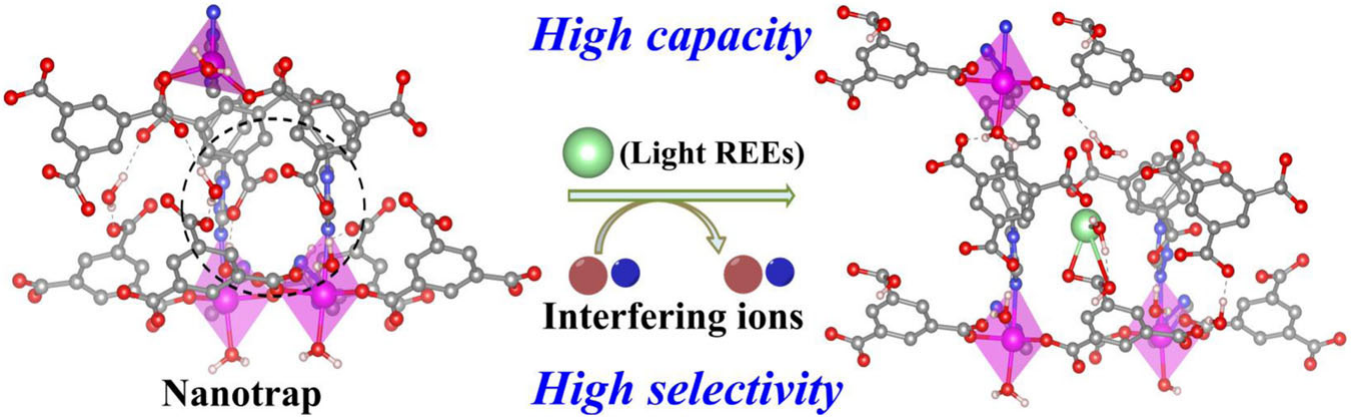
Nanotraps for REE separation. Scheme for REE selectively separation by nanotraps in MOF.

To explore the validity of this hypothesis, we synthesized a new 2D metal-organic framework (termed NCU-1) that features densely uncoordinated carboxyl groups and triazole N atoms, thus providing an REE-philic environment. Owing to the two-fold interpenetration, NCU-1 forms numerous small cavities (3.2 Å) that better match with the size/shape of light REE ions. Therefore, the synergistic effect of suitable apertures and multipoint functional sites in unique REE nanotraps results in a rare high light REE ions uptake and high separation factor for light/heavy REE ions over a single step (Fig. 1). The breakthrough experiments revealed that REE nanotraps could efficiently and selectively separate REE ions from mine tailings over a single step. Thereby, this work provides a powerful approach to obtain high-purity REEs.

## Results

### Synthesis and structure description of NCU-1

**Fig. 2:**
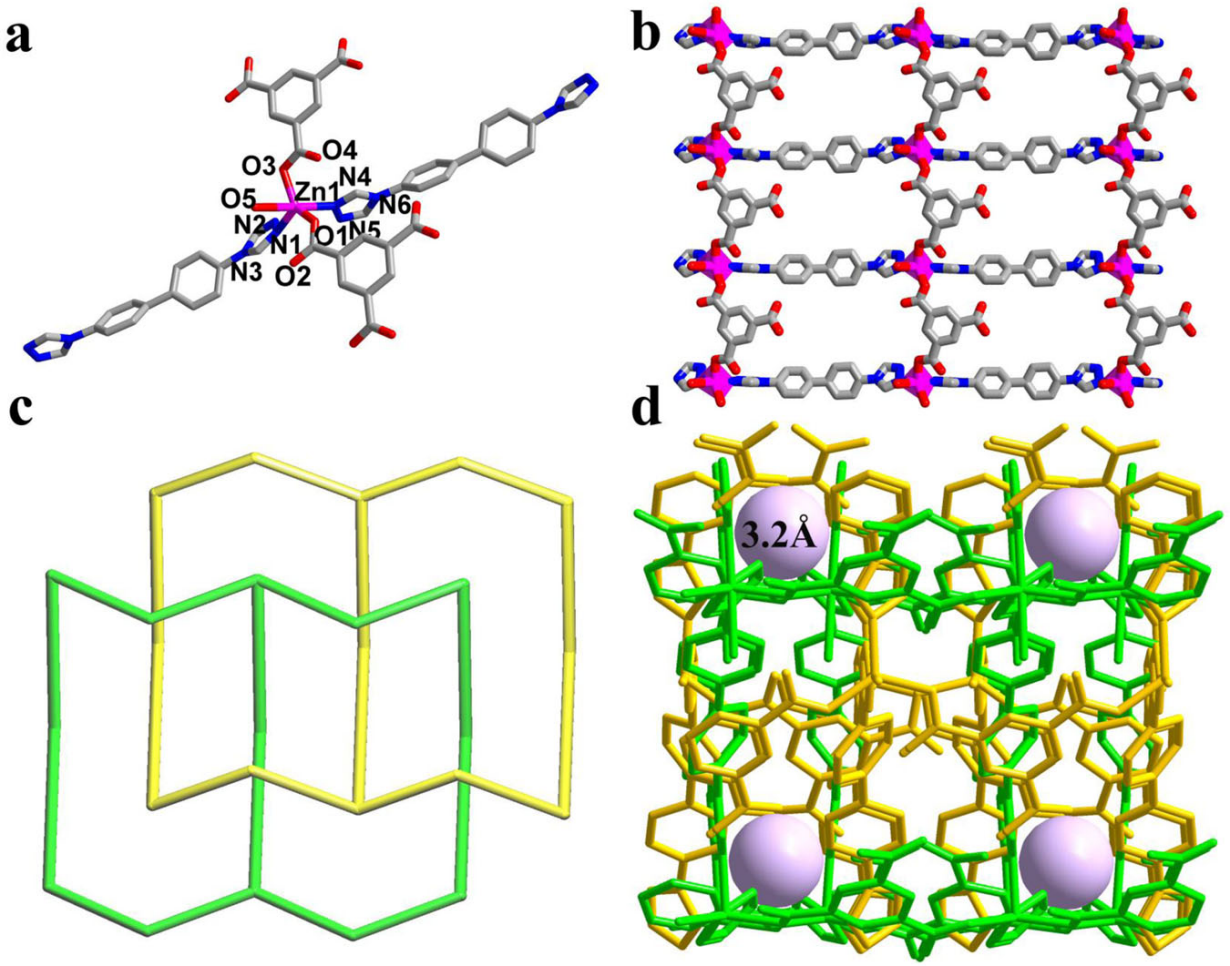
Crystal structure of NCU-1. **a** The coordination environment of Zn^2+^. **b** The 2D extended framework of NCU-1. **c** The simplified topological structure of 2-fold interpenetration (different colors represent different single sets of the interpenetration). **d** The two fold-interpenetrated structure of NCU-1 produces numerous square small pockets. (Zn, magenta; C, dark gray; N, blue; O, red).

The light brown plate crystal of NCU-1 crystallizes in the orthorhombic space group, Pna2_1_ (Supplementary Table 1). The morphology of NCU-1 and its size was studied by SEM, which showed bulk crystals of different sizes (Supplementary Fig. 1). Each Zn^2+^ is 5-coordinated and binds to two DTB ligands, two BTC, and one water molecule (Fig. 2a). Viewed from the *a* axis, rectangular windows with a size of 9.9 Å × 5.8 Å can be observed (Fig. 2b and Supplementary Fig. 2). Owing to the two-fold interpenetration, NCU-1 features numerous small square pockets decorated by multipoint functional sites (Fig. 2c). Each of such small pockets with a size of 3.2 Å is constructed by triazole rings and uncoordinated carboxylate groups that better match with the size/shape of the larger light REE ions (Fig. 2d and Supplementary Fig. 3). The PXRD pattern is consistent with that of the simulated one, indicating the phase purity of NCU-1 (Supplementary Fig. 4). Meanwhile, the chemical stabilities of NCU-1 were investigated upon treatments in various pH values ranging from 2 to 13. The crystallinity of this treated NCU-1 was still retained as evidenced by the PXRD patterns (Supplementary Fig. 5). Furthermore, according to the TG analysis, the NCU-1 showed high thermal stability up to 320 °C (Supplementary Fig. 6). These results suggest that NCU-1 exhibits good stability in harsh conditions, which is highly desirable for capturing and separating of REEs from mine tailings.

### Sorption isotherm analysis

**Fig. 3:**
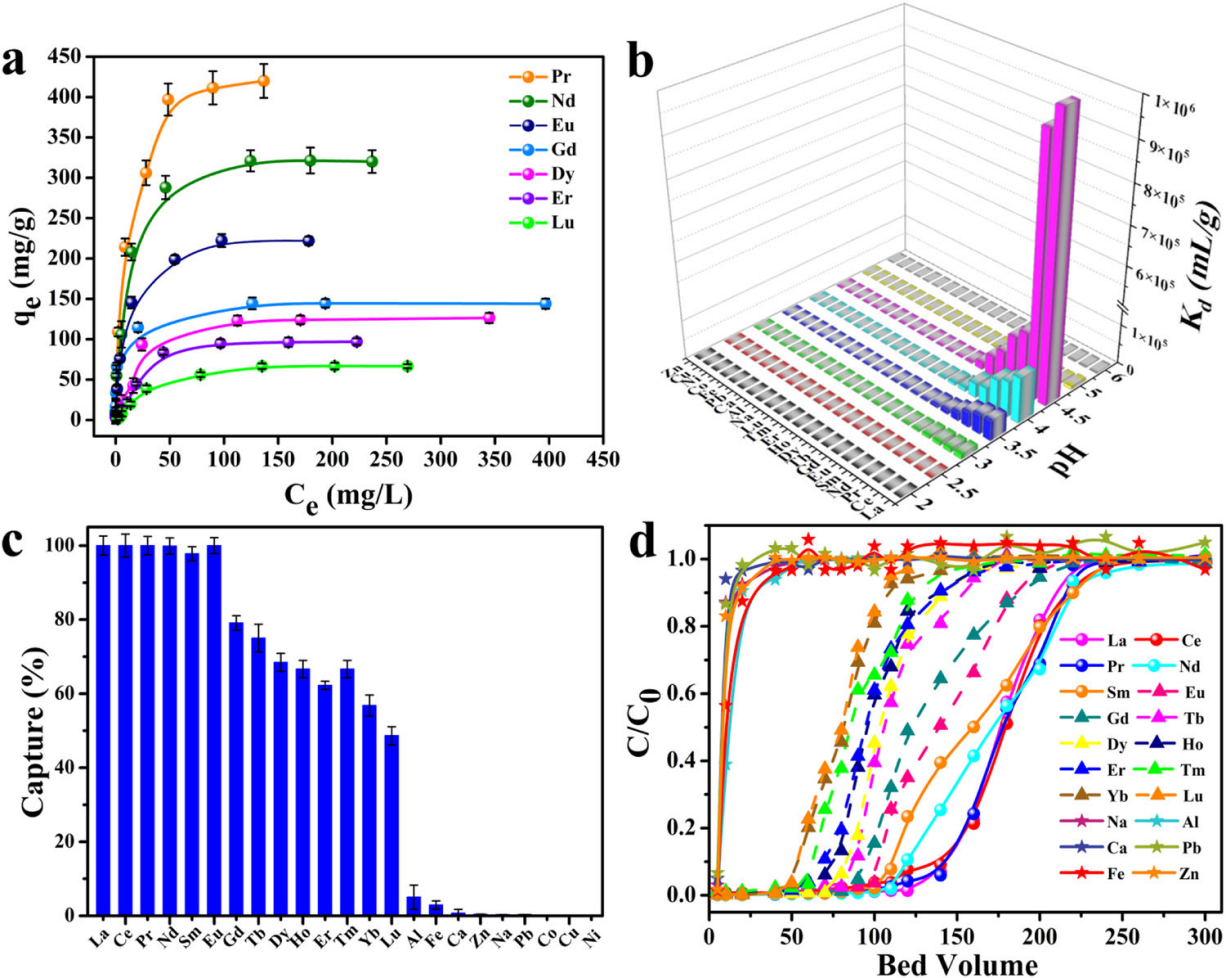
Sorption experiment results of NCU-1. **a** Equilibrium data for Pr^3+^, Nd^3+^, Gd^3+^, and Dy^3+^ adsorption of NCU-1 and fitted with the Langmuir isotherm models. **b** *K*_*d*_ values of metal ions captured by NCU-1 at various pH values from 2.0 to 6.0. **c** NCU-1 capture efficiency of metal ions in mine tailings collected from Ganzhou city. **d** Mine tailing breakthrough curves of metal ions. Error bars represent S.D. *n* = 3 independent experiments.

The adsorption isotherms of Pr^3+^, Nd^3+^, Eu^3+^, Gd^3+^, Dy^3+^, Er^3+^, and Lu^3+^ (Ln^3+^, Ln(NO_3_)_3_·6H_2_O) were explored at pH 4.5 as representative models of REE ions due to their abundance in mine tailings collected from Ganzhou city, Jiangxi province, a well-known REEs industry center in China (Supplementary Figs. 7-19 and Supplementary Table 2). The results showed that the seven Ln^3+^ were fitted well with the Langmuir adsorption model, and the maximum capacity (q_m_) of Pr^3+^, Nd^3+^, Eu^3+^, Gd^3+^, Dy^3+^, Er^3+^, and Lu^3+^ were analyzed by ICP-MS were 420, 310, 221, 140, 126, 97, and 66 mg/g, respectively (Fig. 3a, Supplementary Figs. 20-26, and Supplementary Table 3). This greatly exceeds the capacity of most current adsorbents, such as CA@Fe_3_O_4_ NPs^35^, KIT-6-1,3-PDDA^36^, MFC-O^37^, MIL-101-DGA^38^, and PEI800/ES-1/2.1^39^. Energy dispersive X-ray spectroscopy (EDS) mapping analysis confirms that Pr^3+^, Nd^3+^, Gd^3+^, and Dy^3+^ entered the structures of the material with a uniform distribution throughout the samples (Supplementary Fig. 27). In X-ray photoelectron spectroscopy (XPS) (Supplementary Figs. 28-31), the characteristic peaks of Pr^3+^, Nd^3+^, Eu^3+^, and Er^3+^ were clearly found after adsorption of Pr^3+^, Nd^3+^, Eu^3+^, and Er^3+^, respectively. As depicted in Supplementary Fig. 32, after capturing Pr^3+^, the XRD of NCU-1 shows the shift of some peak sites as compared to the pristine one, which can be attributed to the adsorption of Pr^3+^ on the pores of NCU-1 and interacted with them.

### Sorption kinetics analysis

The kinetics of NCU-1 for individual Pr^3+^, Nd^3+^, Eu^3+^, Gd^3+^, Dy^3+^, Er^3+^, and Lu^3+^ adsorption were then investigated (Supplementary Figs. 33-46 and Supplementary Table 4). The REE ions were rapidly captured by NCU-1, all of which reached equilibrium within 1 h, much shorter than those of the other adsorbents. For example, magnetite@MOF^40^ and hydroxyl-decorated flexible MOF^29^, require longer than 2–4 h to reach equilibrium. Additionally, for any sorbents, recyclability is crucial for practical implementation. Significantly, the adsorption processes were fully reversible as reflected by the fact that the loaded REE ions can be washed off by diluted HNO_3_ at pH 3 with maintained capture efficiency for at least four cycles of sorption/desorption (Supplementary Figs. 47-50). After the fourth cycle, the crystallinity of NCU-1 was maintained, as confirmed by PXRD studies (Supplementary Fig. 51). In addition, the morphology of the fourth reuse of NCU-1 is almost unchanged (Supplementary Fig. 52). The results indicate that NCU-1 can serve robustly towards highly efficient REEs recovery and affirm the judicious choice of the MOF for affording selective trapping REEs.

### Selectivity

The selectivity of REE ions is a key parameter for evaluating adsorbents. Thereby, the distribution coefficient (*K*_*d*_) as a barometer for selectivity was also tested. NCU-1 was dispersed in the mixed solutions containing REE ions and interfering ions with different pH values ranging from 2.0 to 6.0 (Fig. 3b). The *K*_*d*_ of NCU-1 to REEs increases rapidly and reaches the maximum when the pH increases from 2.0 to 4.5, and then decreases to 6.0 (Supplementary Table 5). Compared to transition metal ions and alkaline-earth metal ions, the *K*_*d*_ of NCU-1 to REE ions is 3–4 orders of magnitude higher, suggesting a clear advantage of NCU-1 in selective capture of REE ions. Remarkably, the *K*_*d*_ to various rare-earth ions differs significantly at pH 4.5, which induces that the optimal separation selectivity for REEs can be achieved. Generally, a material is considered an excellent adsorbent if *K*_*d*_ is over 10^4 ^mL g^-1^. Particularly, *K*_*d*_^Gd-La^ was up to 1.6 × 10^4^–1.0 × 10^6 ^mL g^-1^ at pH 4.5. Such high *K*_*d*_ indicates that NCU-1 has an obviously higher affinity toward light REE ions.

### Tailings treatment

Encouraged by the excellent performance of NCU-1 for extraction and separation of REE ions, we evaluated the ability of NCU-1 to capture and separate REE ions from mine tailing collected from Ganzhou city. The natural rare-earth tailing sample was filtered through a 0.22 µm filter for ICP-MS analysis. The results show that the concentrations of rare earth elements in the tailing are low about 0.09–0.92 ppm, and the competitive ions concentration of Al (8.15 ppm) is about 10 times compared to those of the REEs (Supplementary Table 2), making the REEs adsorption and separation a great challenging task. Except for competing ions, the NCU-1 could effectively selective capture all REE ions from the natural mine tailing (Fig. 3c), manifesting it is a good platform for adsorption and separation of REEs from mine tailings. As an industrially relevant performance test, experimental breakthrough studies were performed to evaluate the actual separation ability of NCU-1 (Supplementary Fig. 53). The competitive ions occurs first at 10 bed volumes and REE ions occur after 50 bed volumes (Fig. 3d and Supplementary Fig. 54), indicating NCU-1 has an excellent separation effect on REE ions from competitive ions. It is worth noting that compared to heavy REE ions, light REE ions have even higher uptakes and larger breakthrough bed volumes. For instance, the breakthrough bed volume of Pr^3+^ is 150, which is larger than that of Dy^3+^ (80) (Supplementary Fig. 55). The results illustrate that NCU-1 is a promising candidate for separation and obtaining high purity REE ions from mine tailing over a single step.

### Separation mechanism

The FT-IR and XPS were applied to evaluate the structure-performance relationship of NCU-1 in the effective capture of REE ions. After adsorption Pr^3+^ and Nd^3+^, the signal of -COOH shows a large blue shift of 22.1 cm^-1^ and 20.0 cm^-1^, respectively (from 1574.5 cm^-1^ to 1552.4 cm^-1^ and 1554.5 cm^-1^)^26^, and the peak of N atoms from triazole has a 12.7 cm^-1^ and 11 cm^-1^ (from 1519.2 cm^-1^ to 1531.9 cm^-1^ for Pr^3+^, and 1530.2 cm^-1^ for Nd^3+^, respectively) redshift (Supplementary Figs. 56 and 57), illustrating that the robust interaction between Ln^3+^ and carboxyl groups and triazole groups in NCU-1. As shown in Supplementary Fig. 28, the character peak at 933.95 eV of NCU-1-Pr ascribed to Pr 3*d*^5/2^ demonstrated that Pr^3+^ was successfully adsorbed into the NCU-1. After adsorption of Nd^3+^, distinctive Nd 3*d*^5/2^ and 3*d*^3/2^ peaks appeared at 982.95 and 1005.59 eV, which had a great blue shift of 0.85 eV compared to the signal (BE, 983.70 eV) of Nd 3*d*^5/2^ in Nd(NO_3_)_3_^41^, indicating that Nd^3+^ was successfully loaded onto the NCU-1 and had strong interaction between them (Supplementary Fig. 29). In the O 1 *s* high-resolution spectrum, the signals at 531.36 eV and 533.01 eV are assigned to -COOH and water, respectively (Supplementary Fig. 58). Obviously, the peak at 531.36 eV of –COOH in NCU-1 has a significant positive shift by 0.60 eV and 0.18 eV after capturing Pr^3+^ and Nd^3+^ respectively, proving strong interactions between Ln^3+^ and the carboxyl in NCU-1, which is consistent with the results of the C 1 *s* high-resolution spectrum (Supplementary Figs. 59-63). In addition, the binding energies at 533.01 eV of NCU-1 shifted by 0.49 eV and 0.37 eV to higher binding energy after extraction of Pr^3+^ and Nd^3+^, respectively. These results suggest that the water molecules have also participated in the coordination of rare-earth ions. Comparing the N 1 *s* peaks in the before and after adsorption Ln^3+^ of two different samples (Supplementary Figs. 64-66), the -NH (399.98 eV) peak shifted by 0.40 eV and 0.36 eV to lower binding energy after extracting Pr^3+^ and Nd^3+^, respectively. After adsorption of Pr^3+^ and Nd^3+^, the -C = N- peaks at 401.44 eV were changed to 401.28 and 401.36 eV, respectively. This implies that triazolium nitrogen atoms in NCU-1 were also involved in the coordination of Ln^3+^. In conclusion, the results show that the high adsorption toward REE ions of NCU-1 is ascribed to the strong coordination ability between the REE ions and carboxyl groups, lattice water molecules, and triazolium nitrogen atoms in the host.

**Fig. 4:**
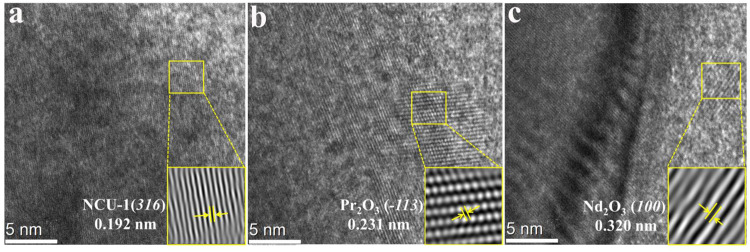
Cryo-EM images of NCU-1 and after adsorption REEs. Cryo-EM images of **a** NCU-1, **b** NCU-1-Pr, and **c** NCU-1-Nd.

**Fig. 5:**
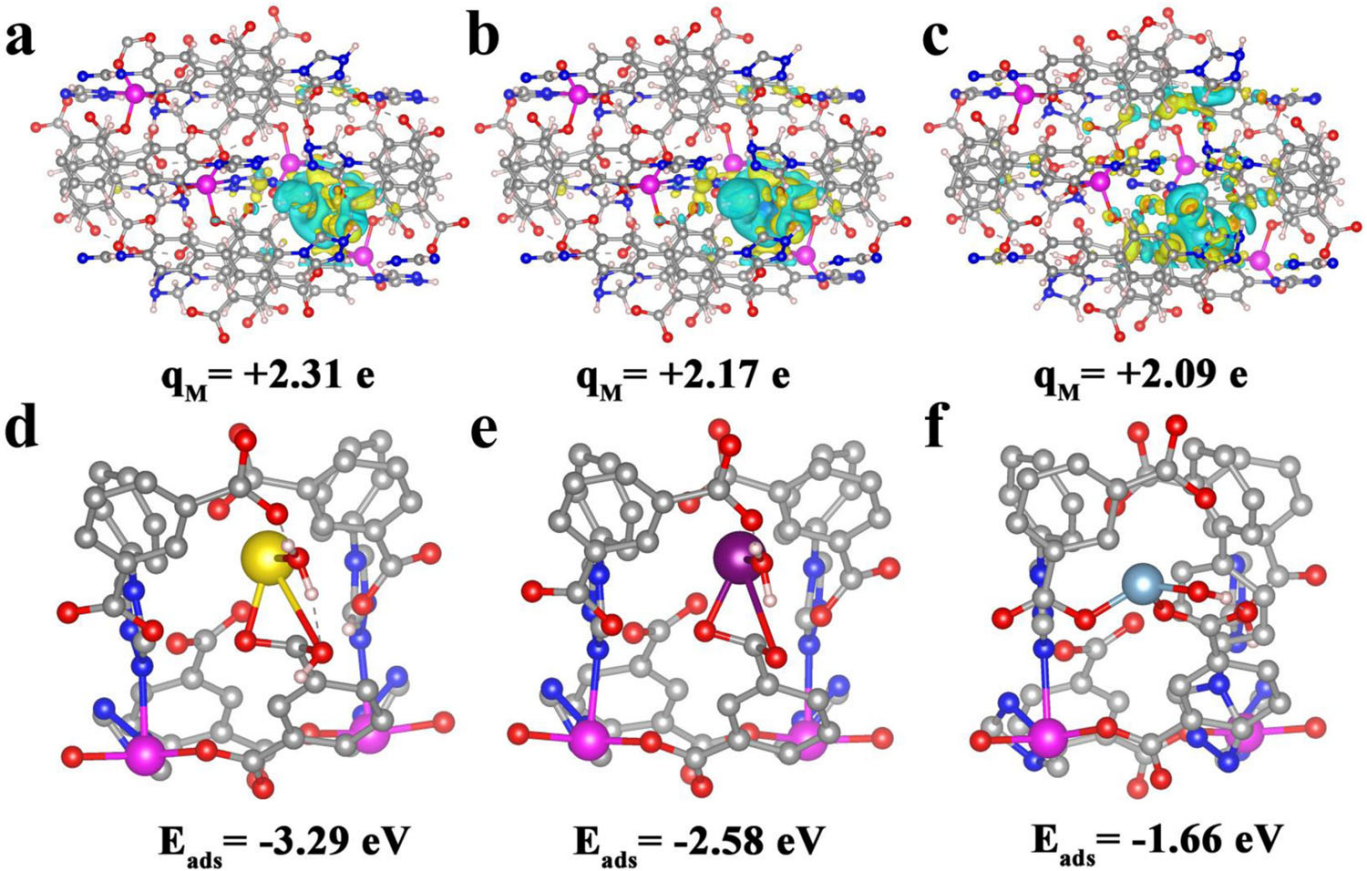
The first principle computations and density functional theory analysis. Differential charge distribution of NCU-1 configuration after adsorption with **a** Pr^3+^, **b** Dy^3+^, and **c** Al^3+^ with the corresponding Bader charges. The Bader charge on the metal ion is defined as q_M_. Optimized structures of NCU-1 and the adsorption energy of metal ions **d** Pr^3+^, **e** Dy^3+^, and **f** Al^3+^ (Pr^3+^, yellow; Dy^3+^, purple; Al^3+^, light blue).

To examine the crystal structures of the NCU-1-REEs ions in greater detail, we performed additional investigations using Cryo-Electron Microscopy (Cryo-EM). As shown in Fig. 4a, the NCU-1 has distinct lattice fringes with a spacing of 0.192, corresponding to the (*316*) crystal planes. The Cryo-EM image of NCU-1-Pr and NCU-1-Nd with obvious lattice fringes evidenced the highly crystalline nature after adsorbing REEs. Among these, the lattice spacings of 0.231 nm and 0.320 nm, correspond to the (*-113*) crystal planes of Pr_2_O_3_ and (*100*) of Nd_2_O_3_, respectively^42,43^ (Fig. 4b, c). These results suggested that the rare earth ions may be trapped by REE nanotrap and interact with the oxygen atoms of carboxylic acid in NCU-1 to form oxide species.

elucidate the origin of the observed high selectivity of the NCU-1 for REE ions, the first principle calculations were carried out between metal ions and the NCU-1 configurations. Al^3+^, Dy^3+^, and Pr^3+^ were selected as the representative interfering ions, heavy REEs ions, and light REE ions, respectively. Figure 5a–c show the differential charge density (DCD) maps of NCU-1 configurations after adsorption Al^3+^, Dy^3+^, and Pr^3+^, respectively. Obviously, charge redistribution occurs at metal ions and NCU-1, and the negatively charged regions (yellow) with excess electrons accumulate around the O atoms and N atoms, which are beneficial to the adsorption of metal ions^44,45^. The Bader charge analysis^46^ shows that the charges on Al^3+^ ion in the NCU-1 configuration are +2.09 e, while +2.17 e for Dy^3+^, and increase to +2.31 e for Pr^3+^. Generally, the larger the Bader charge, the stronger the binding strength between the host and guests^47^. Therefore, the order of the binding strength of NCU-1 with the three ions is as follows: Al^3+^<Dy^3+^<Pr^3+^. It suggests that the selectivity of NCU-1 for light REE ions is higher than those of heavy REE ions and interfering ions, due to the more electrons obtained from the host. Moreover, the density functional theory (DFT) calculation clearly reveals that the primary adsorption sites of metal ions are located at the REE nanotraps (Fig. 5d–f). As for Pr^3+^, two oxygen atoms of the carboxyl group and one water molecule were calculated to coordinate with Pr^3+^ with the bond distances of 1.79 Å, 2.73 Å, and 1.94 Å, respectively (Supplementary Fig. 67). The calculated distance of Pr^3+^···N in NCU-1 is 3.30 Å. In the case of Nd^3+^, it is coordinated to the two oxygen atoms of the carboxyl group and one water molecular with the bond distances of 1.66 Å, 2.76 Å, and 1.86 Å, respectively, and the Nd^3+^···N bond distance is about 3.27 Å (Supplementary Fig. 68). While as for Al^3+^, only the coordination bonds between the carboxyl and water molecular oxygen and Al^3+^ were calculated (Supplementary Fig. 69). The adsorption energies (Eads) of NCU-1 with different metal ions were also calculated to discriminate the adhesion level. The calculated adsorption energy for Al^3+^, Dy^3+^, and Pr^3+^ are -1.66 eV, -2.58 eV and -3.29 eV, respectively. It decreases when the size of the adsorbed ions increases, which is fully consistent with our experimental observations. This is partly because the larger Pr^3+^ (*r* = 0.99 Å) sterically “matches” better to the nanotraps size/shape than Dy^3+^ (*r* = 0.91 Å) and Al^3+^ (*r* = 0.50 Å)^6,17^. Light REE ions within a larger size radius enter the REE nanotraps and form stable complexes with the uncoordinated carboxylic groups and N atoms in the NCU-1^30^. However, competing ions and heavy rare earth ions with a large difference in pore size from NCU-1 have poor binding ability to coordinate with O, which makes the ions easily escape from the pores and achieve high-selectivity separation of light RREs. Therefore, the suitable sizes of the nanotraps and the uncoordinated carboxyl groups and N atoms in nanotraps play a decisive role in the light/heavy REE separation.

### Binary REE ions separation

**Fig. 6:**
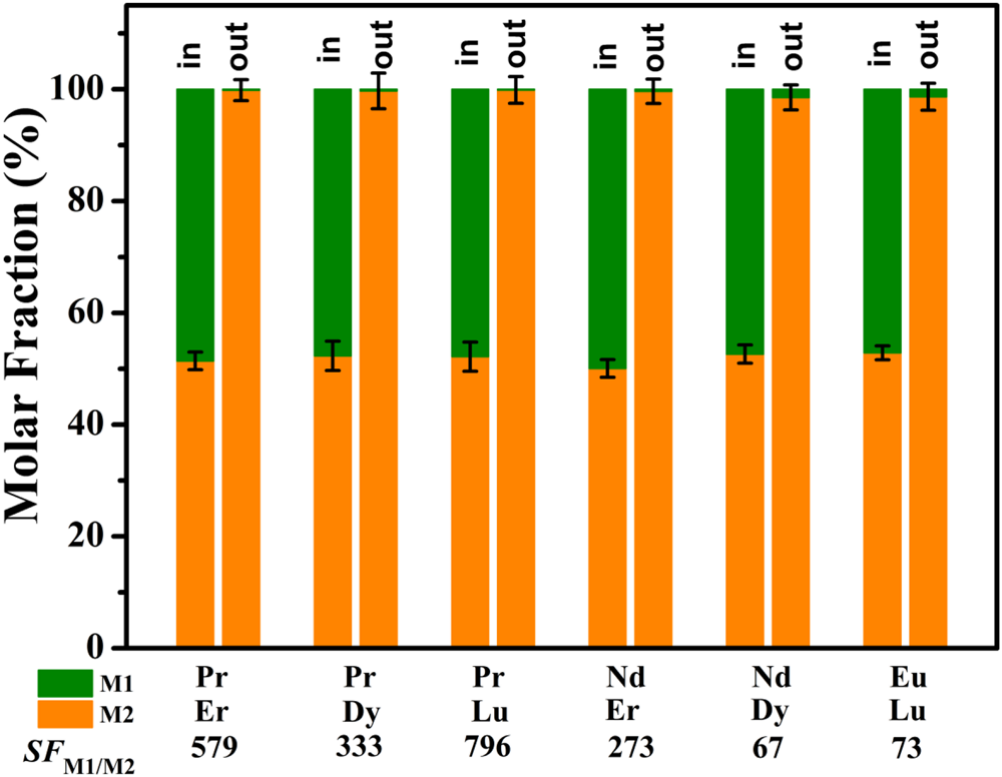
Binary REE ions separation models and results. Selective adsorption of NCU-1 from six combinations of a light REEs M1 and a heavy REEs M2. “In” and “out” indicate the REEs ratio in the original mixed solution and filtrates. Error bars represent S.D. *n* = 3 independent experiments.

Since the affinity of NCU-1 to REE ions exhibits a unique periodic trend with significant differences, the individual REE can be separated from other REEs by the adsorption method (Fig. 3b). As a proof of concept, a series of experiments (6 combinations in total) were conducted to determine its application on individual REE separations. Consequently, NCU-1 was dispersed into binary mixtures of REE ions (M1 and M2) in a 1:1 molar ratio. As shown in Fig. 6, larger Ln^3+^ is selectively adsorbed by NCU-1, and smaller Ln^3+^ almost remained in the solution, indicating that this system successfully separated light/heavy REE mixtures. For a mixtures that contained Nd^3+^ and Dy^3+^, the separation factor (*SF*) reached up to 67, which exceeded those of many materials, for example, TriNOx^3-^ (*SF*_Nd:Dy_ = 8.45 ± 1.62)^4^, Zn-BTC (*SF*_Nd:Dy_ = 30.23)^30^, TRPO (*SF*_Nd:Dy_ < 5)^11^. Especially interesting is the separation factor of 73 observed for Eu^3+^ over Lu^3+^, a value that is high than that of the crystallization extraction method recently developed by Wang et al.^3^. Impressively, the case of Pr/Lu, obtains a high separation factor of 796, which is one of the highest single-step separation factors known for REEs separations^48^. Mixtures that contained Nd: Er also gave a large separation factor of *SF*_Nd: Er_ = 273. Generally, NCU-1 demonstrates a clear and high preference for larger-sized REE ions in the solid adsorption process, and the selectivity increases with the ionic radii difference. High-purity individual rare-earth ions can be obtained through one-step adsorption in this work, which has made a breakthrough process in the separation of REEs.

## Discussion

In conclusion, we report a unique REE nanotrap featuring dense uncoordinated carboxyl groups and triazole N atoms and suitable pore sizes in NCU-1 that can induce strong interaction with light REE ions. Thereby the NCU-1 affords outstanding light REEs adsorption and separation performance for potential use in capturing REEs from mine tailings. Notably, the high response to light REE is firstly realized by simple adsorption progress, which could obtain high purity individual REEs over a single step. Not only does this work report a promising platform that offers a solution to the long-term challenge of REEs recovery from complex wastewater systems, but it also sheds light on the rational design of metal framework materials for applications in the field of REE purification.

## Methods

### Materials

4,4-di(4H-1,2,4-triazol-4-yl)-1,1-biphenyl (DTB) and trimesic acid (BTC) were purchased from Jilin Chinese Academy of Sciences-Yanshen Technology Co., Ltd. Zn(NO_3_)·6H_2_O and trivalent lanthanide nitrates were purchased from Energy Chemical Co., Ltd. Acetonitrile (MeCN) and ethanol were purchased from Sinopharm Chemical Reagent Co., Ltd. Ultrapure water was prepared from the Millipore system (18.25 MΩ cm). All the purchased reagents were of analytical grade and used without further purification.

### Synthesis of NCU-1

To a 20 mL PTFE reactor, trimesic acid (21.0 mg, 0.10 mmol), 4,4-di(4H-1,2,4-triazol-4-yl)-1,1-biphenyl (28.8 mg, 0.10 mmol), Zn(NO_3_)·6H_2_O (29.5 mg, 0.1 mmol), MeCN 2 mL, and water 8 mL were added. The mixture was sealed and heated 140 °C for 3 days, followed by slow cooling at a rate of 5 °C/h to room temperature, and light brown crystals for X-ray analysis were collected and dried in air.

### Supplementary information

Supplementary Information

## Data Availability

The data that support the findings of this paper are available in the paper and supplementary information files. The X-ray crystallographic data for the structure reported in this article has been deposited at the Cambridge Crystallographic Data Centre (CCDC) with the number CCDC 2141439. Any further relevant data are available from the corresponding authors upon request.
