# Supplementary material for: Rationally designed nanotrap structures for efficient separation of rare earth elements over a single step
Source: Nat Commun. 2024 Feb 20;15:1558. doi: 10.1038/s41467-024-45810-1 (PMC10879098; doi:10.1038/s41467-024-45810-1)
Supplement: Supplementary file 1 — Supplementary Information [file 41467_2024_45810_MOESM1_ESM.pdf]

Supplementary Information

Rationally designed nanotrap structures for efficient separation of rare earth elements over a single step

Qing-hua Hu[1,2,3], An-Min Song[2], Xin Gao[2], Yu-zhen Shi[2], Wei Jiang[2], Ru-Ping Liang[2]* & Jian-Ding Qiu[1,2]*

[1]State Key Laboratory of Nuclear Resources and Environment, East China University of Technology, Nanchang 330013, China

[2]School of Chemistry and Chemical Engineering, Nanchang University, Nanchang 330031, China

[3]School of Chemistry and Chemical Engineering, Jinggangshan University, Jian 343009, China

*Corresponding authors. e-mail: rpliang@ncu.edu.cn; jdqiu@ncu.edu.cn

Characterizations and Methods

Instruments. Fourier-transform infrared (FT-IR) spectra were recorded on a Nicolet Impact 410 FT-IR spectrometer. The morphology of the material was imaged by a scanning electron microscope (SEM, JEM-2010, JEOL). Powder X-ray diffraction (PXRD) data of the crystals were collected on a Bruker AXS D8 Advance A25 Powder X-ray diffractometer (40 kV, 40 mA) using Cu Kα (λ=1.5406 Å) radiation. X-ray photoelectron spectroscopy (XPS) spectra were performed on a Thermo VG Multilab 2000X with Al Kα irradiation. The thermal property of the NCU-1 was evaluated using an STA PT1600 Linseis thermogravimetric analysis (TGA) instrument over the temperature range of 30 to 800 ℃ under a nitrogen atmosphere with a heating rate of 10 ℃/min. Metal ions concentrations were determined using an iCAP Q inductively coupled plasma mass spectrometry (ICP-MS, Thermo Fisher Scientific, USA). A suitable single crystal of complex was mounted on a loop for the X-ray measurement. Diffraction data were collected on SuperNova (Dual-source) diffractometer equipped with graphic monochromatic Cu Kα radiation (λ = 1.54184 Å) using the CrysAlispro X-ray crystallography data systems at 100 K under a cold nitrogen stream. The structure of the crystal was solved with the ShelXT structure solution program adopting Intrinsic Phasing and refined with the ShelXL refinement package adopting Least Squares minimisation by Olex2.[1,2] The crystal data of NCU-1 is listed in Table S1. Crystallographic data have been deposited at the Cambridge Crystallographic Data Center with reference number CCDC.

Synthesis Scale-Up of NCU-1. A large-scale synthesis of NCU-1 was performed. Trimesic acid (0.840 g, 4 mmol), 4,4-di(4H-1,2,4-triazol-4-yl)-1,1-biphenyl (1.15 g, 4 mmol), $Zn(NO_3)·6H_2O$ (1.18 g, 4 mmol), MeCN 80 mL, and water 320 mL were added into a PTFE reactor. The mixture was sealed and heated at 140 ℃ for 3 days, followed by slow cooling at a rate of 5 ℃/h to room temperature, and light brown crystals for X-ray analysis were collected and dried in air (Yield: 1. 19 g; 80.1%). In addition, the PXRD pattern is consistent with that of the simulated one, indicating that the synthesis of NCU-1 is simple and can be scaled up.

Stability Test. NCU-1 was immersed in different pH (1, 3, 5, 7, 9, 11, 13) solution for 12 h, respectively. The mixture was then filtered and washed with ultra-pure water till the supernatant became neutral and dried under vacuum at 60 ℃. Then, the PXRD patterns were obtained.

Sorption Experiments. The solid-liquid ratio in all experiments was 1.0 g/L. The pH value of the solutions was adjusted to 4.50 with HNO_3 or NaOH aqueous solution. The rare earth tailing samples were collected from the Ganzhou City, Jiangxi Province. The concentrations of REE ions during all the experiments were detected by ICP-MS for extra-low concentrations. All the adsorption experiments were performed under ambient conditions. All experiments were conducted in triplicate, with average values displayed in the graphs.

REE Ions Adsorption Isotherm Experiments. Adsorption experiments were performed according to the reported methods.[3] The Pr^{3+}, Nd^{3+}, Eu^{3+}, Gd^{3+},Dy^{3+}, Er^{3+}, or Lu^{3+} adsorption capacity assay was performed by adding 10 mg NCU-1 into 10 mL of varying the initial concentrations of Pr^{3+}, Nd^{3+}, Eu^{3+}, Gd^{3+}, Dy^{3+}, Er^{3+}, or Lu^{3+} (10, 20, 50, 100, 150, 200, 250, 300, 400, 500, 600, 800, 1000 mg/L) solution with a pH of 4.50 with moderate magnetic stirring, respectively. After achieving equilibrium, the treated solution was filtered through a 0.22 μm membrane filter, and the filtrate was collected and analyzed by using ICP-MS to determine the remaining REE ions content. The adsorption capacity was calculated based on equation (1). The theoretical maximum adsorption capacity can be calculated by the Langmuir and Freundlich adsorption isotherms, as depicted in equations (2) and (3).

$$q_e = \frac{(C_0 - C_e)V}{m} \qquad (1)$$

$$\frac{C_e}{q_e} = \frac{1}{q_m k_L} + \frac{C_e}{q_m} \qquad (2)$$

$$\ln q_e = \ln k_F + \frac{1}{n} \ln C_e \qquad (3)$$

where V is the volume of the treated solution (L), m is the amount of used adsorbent (g), C_o is the initial concentration of REE ions (mg/L), and C_e is the equilibrium concentration of REE ions (mg/L), respectively. q_e (mg/g) is the equilibrium

adsorption capacity. q_m (mg/g) is the maximum sorption capacity, k_L is a constant indirectly related to sorption capacity and energy of sorption (L/mg), which characterizes the affinity of the adsorbate with the adsorbent. k_F and n are the Freundlich constants related to the sorption capacity and the sorption intensity, respectively.

REE Ions Adsorption Kinetics Experiments. 10 mg of NCU-1 was added into 10 mL aqueous solutions of the initial concentrations of Pr^{3+}, Nd^{3+}, Eu^{3+}, Gd^{3+}, Dy^{3+}, Er^{3+}, or Lu^{3+} (5 mg/L). Under magnetic stirring, the resulting mixture was stirred for the desired contact time (5 min, 10 min, 20 min, 30 min, 40 min, 50 min, 60 min, 120 min, and 240 min), then using 0.22 um nylon membrane filter for ICP-MS detection. The capture percentage was calculated based on equation (4). Pseudo-first-order model and pseudo-second-order model are usually used for the sorption kinetics data fitting. The formulas were expressed in equations (5) and (6), respectively.

$$\text{Capture\%} = \frac{(C_0 - C_t)}{C_0} \times 100\% \quad (4)$$

$$\ln(q_e - q_t) = \ln q_e - k_1 t \quad (5)$$

$$\frac{t}{q_t} = \frac{1}{k_2 q_e^2} + \frac{t}{q_e} \quad (6)$$

where q_e and q_t are the adsorption capacity at equilibrium and time t, k_1 (g mg^{-1} min^{-1}) and k_2 (g mg^{-1} min^{-1}) are the rate constants of pseudo-first-order and pseudo-second-order, respectively. The pseudo-first-order linear plot can be obtained by plotting ln (q_e-q_t) versus t, and the pseudo-second-order linear plot can be obtained by plotting t/q_t against t.

pH-dependent Adsorption Experiments. 10 mg NCU-1 adsorption material was added to a 10 mL aqueous solution containing 5 mg/L REE ions (La-Lu) and 5 mg/L competing transition metal and alkali metal ions (Na^{+}, Al^{3+}, Ca^{2+}, Fe^{3+}, Co^{2+}, Ni^{2+}, Cu^{2+}, Zn^{2+}, Pb^{2+}) with varying pH (2.0, 2.5, 3.0, 3.5, 4.0, 4.5, 5.0, 6.0), and the solution pH was adjusted as required using NaOH and HNO_3. The mixtures were shaken at a rate of 120 rpm for 6 h, and separated with a 0.22 μm nylon membrane filter for ICP-MS analysis. The affinity and selectivity of NCU-1 to REE ions can be

detected by the distribution coefficient (K_d), as illustrated in equations (7).

$$K_d = \frac{(C_0 - C_e)V}{C_e m} \qquad (7)$$

Recyclability Test. After one run of adsorption, the adsorbents were regenerated by treatment with the elution solution of HNO_3 (pH 3) solution and reused for another adsorption experiment. For 100 mg adsorbents, a 100 mL elution solution was used to elute the binding REE ions (Pr^{3+}, Nd^{3+}, Gd^{3+}, or Dy^{3+}) for 6 h at room temperature. The resulting suspension was filtered and washed with ultra-pure water till the supernatant became neutral. After being dried under vacuum, the resultant material was used for another adsorption experiment. It was found that after four consecutive cycles NCU-1 still showed excellent REE uptake.

Binary Lanthanide Separation by NCU-1. Pr/Er separation: 10 mg NCU-1 was charged into a 10 mL mixture solution containing $Pr(NO_3)_3·6H_2O$ (0.03 mmol), $Er(NO_3)_3·6H_2O$ (0.03 mmol). The mixtures were shaken at a rate of 120 rpm for 6 h, and separated with a 0.22 μm nylon membrane filter for ICP-MS analysis. The other five binary lanthanide separations of Pr/Dy, Pr/Lu, Nd/Er, Nd/Dy, and Eu/Lu were under the same procedure with Pr/Er binary separation. Separation factors (*SF*) are calculated using the following equation (8).[4] All the molar fraction (*B%*) value was calculated from the separation factor, as shown in equation (9).[5]

$$\mathrm{SF} = \frac{K_d^1}{K_d^2} \qquad (8)$$

$$\mathrm{B\%} = \frac{SF}{1+SF} \times 100\% \qquad (9)$$

The Rare Earth Tailing and Experimental Breakthrough Studies. The natural rare earth tailing sample was collected from Ganzhou city, Jiangxi province, and filtered through a 0.22 μm filter to remove insoluble particles and microorganisms. 10 mg adsorbent was dispersed into the treated natural rare earth tailing sample. The mixtures were shaken at a rate of 120 rpm for 6 h, and separated with a 0.22 μm nylon membrane filter for ICP-MS analysis.

500 mg crystalline products of NCU-1 as the stationary phase were filled in a glass column, the inside diameter of the column is 9 mm, and the treated mine tailing

was collected from Ganzhou city, as mobile phase under ambient pressure (Figure S9). The solutions with metal ions were controlled to slowly pass through the column and collected at the bottom in a bottle for ICP-MS analysis.

Theoretical Calculations. First-principles calculations were carried out using density functional theory (DFT) with generalized gradient approximation (GGA) of Perdew-Burke-Ernzerhof (PBE) implemented in the Vienna Ab-Initio Simulation Package (VASP).[6,7] The valence electronic states were expanded on the basis of plane waves with the core-valence interaction represented using the projector augmented plane wave (PAW) approach[8] and a cutoff of 520 eV. A Γ-centered k-mesh of 1 × 1 × 1 was used for the surface calculations. Convergence is achieved when the forces acting on ions become smaller than 0.02 eV/Å.

The adsorption energies of metal ions on NCU-1 were defined by the equation (10).

$$E_{ads} = E_{M^*} - E_{NCU-1} - E_M \qquad (10)$$

where E_{M*} is the total energy of the Metal-adsorbed NCU-1; $E_{NCU\text{-}1}$ is the energy of the NCU-1, and E_M is the energy of isolated metal ions such as Al^{3+}, Dy^{3+}, and Pr^{3+}.

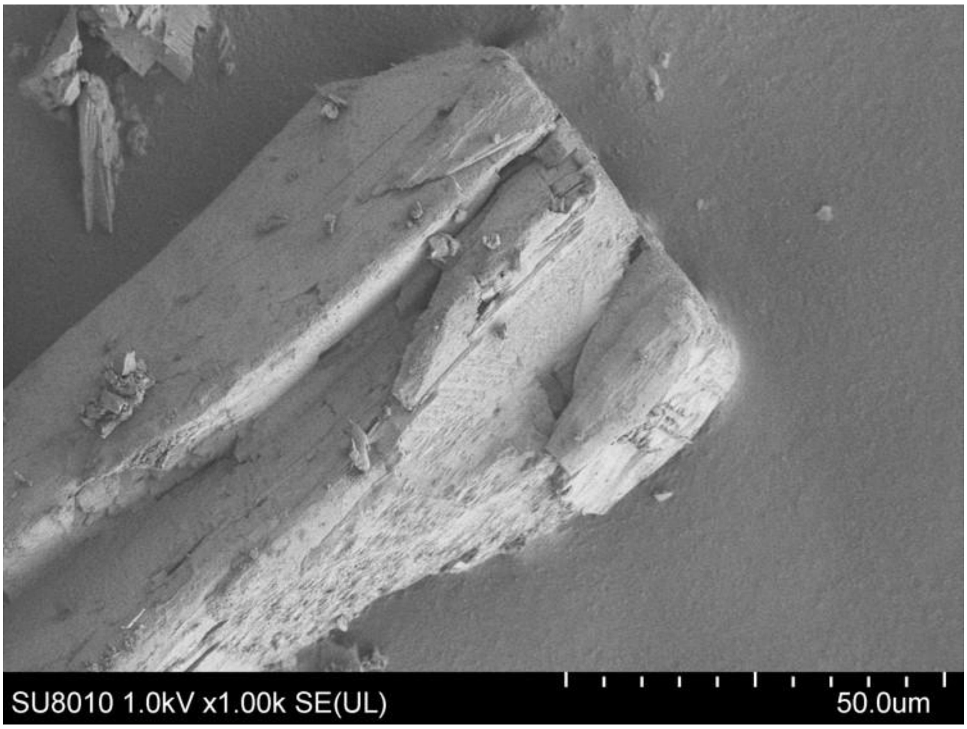

Supplementary Fig. 1丨SEM image of NCU-1.

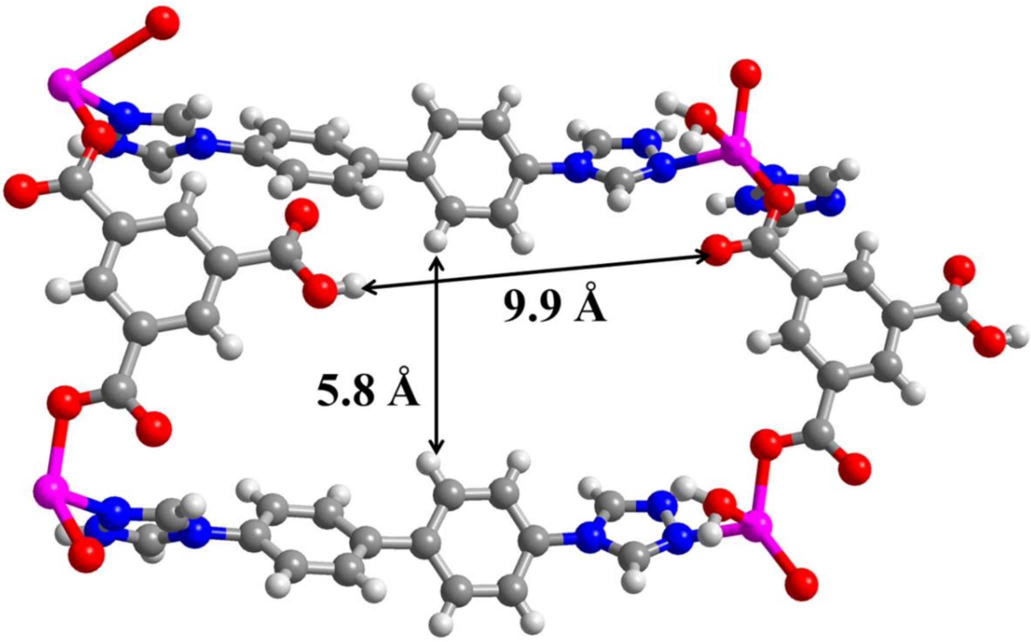

Supplementary Fig. 2丨Diagram of the square windows formed by four Zn^{2+} ions, two trimesic acids, and DTB ligands.

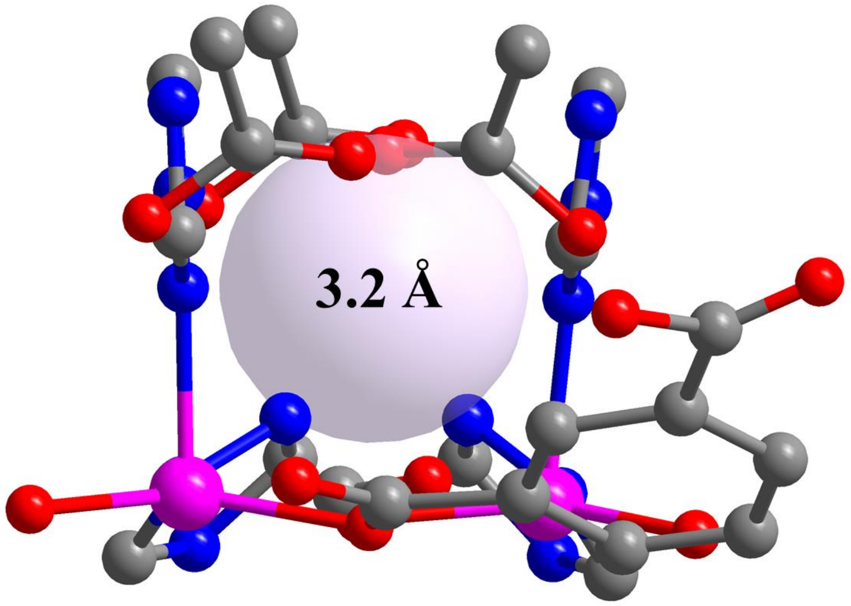

Supplementary Fig. 3 | Depiction of one pocket with a diameter of 3.2 Å constructed by triazole rings and carboxylate groups (Zn, magenta; C, dark gray; N, blue; O, red).

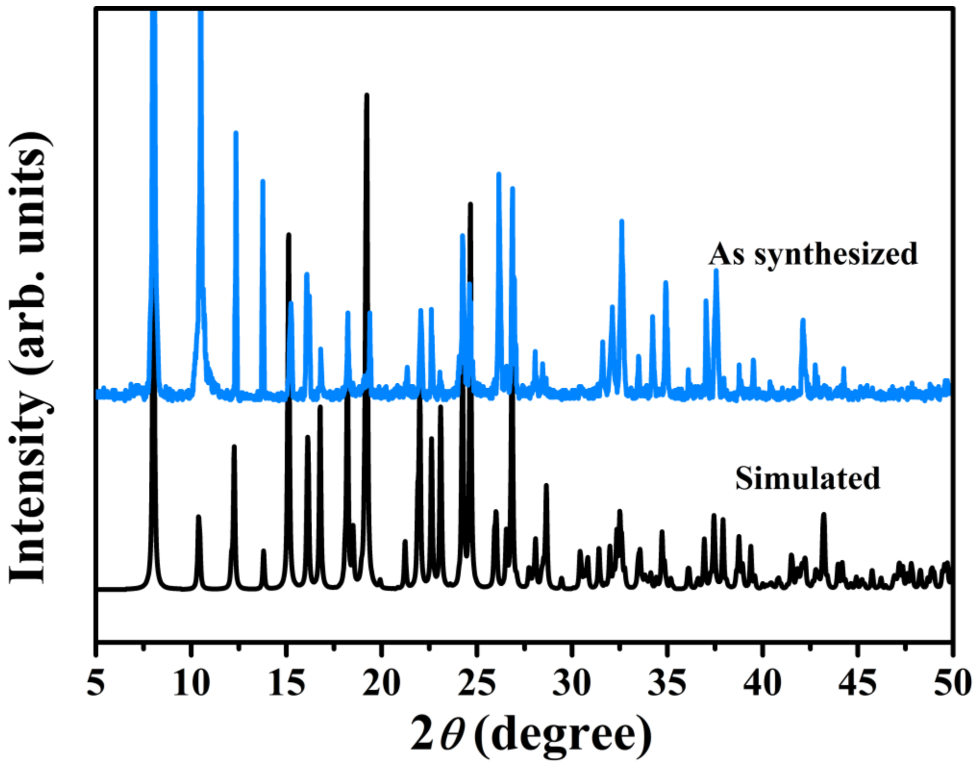

Supplementary Fig. 4 | PXRD patterns of the simulated NCU-1 and as synthesized samples. The positions of the diffraction peaks of the two are basically the same, indicating that NCU-1 is a pure phase.

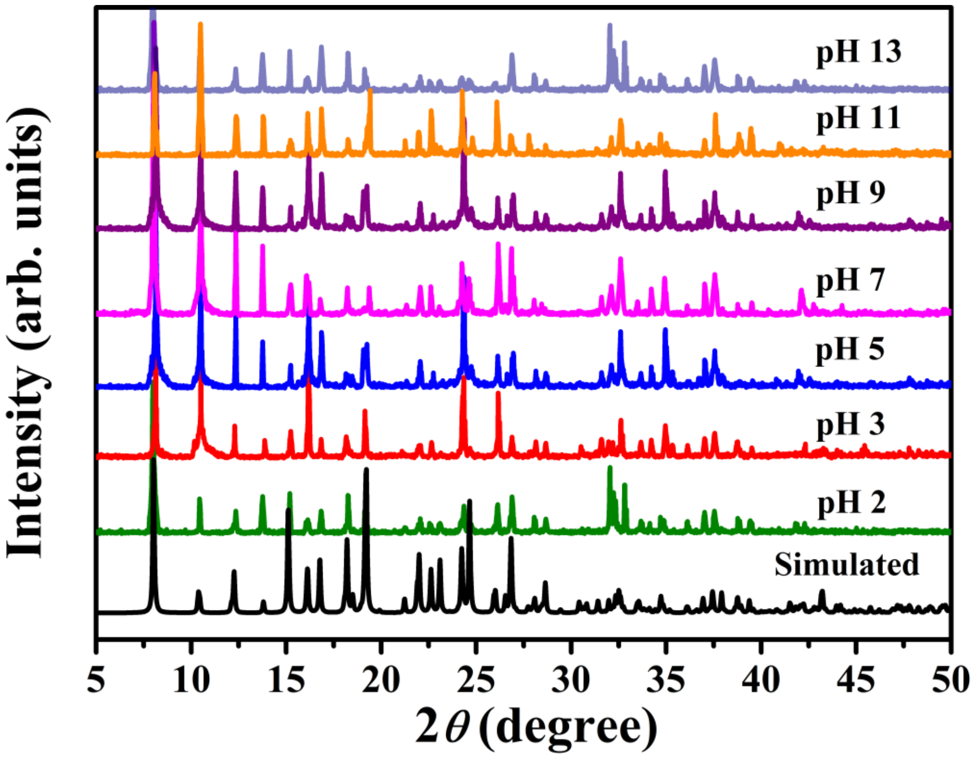

Supplementary Fig. 5 | PXRD patterns of NCU-1 after immersion in aqueous solutions with different pH values ranging from 2 to 13 for 12 h.

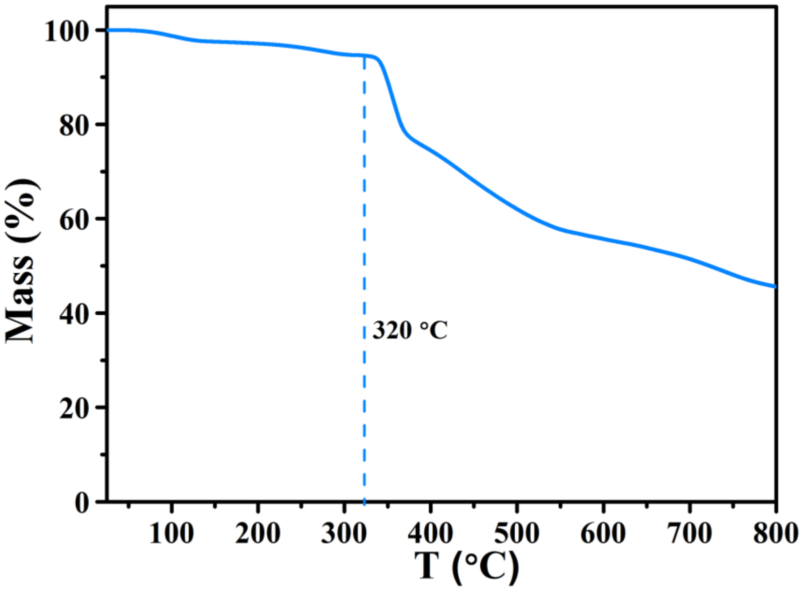

Supplementary Fig. 6 | The thermogravimetric analysis of NCU-1.

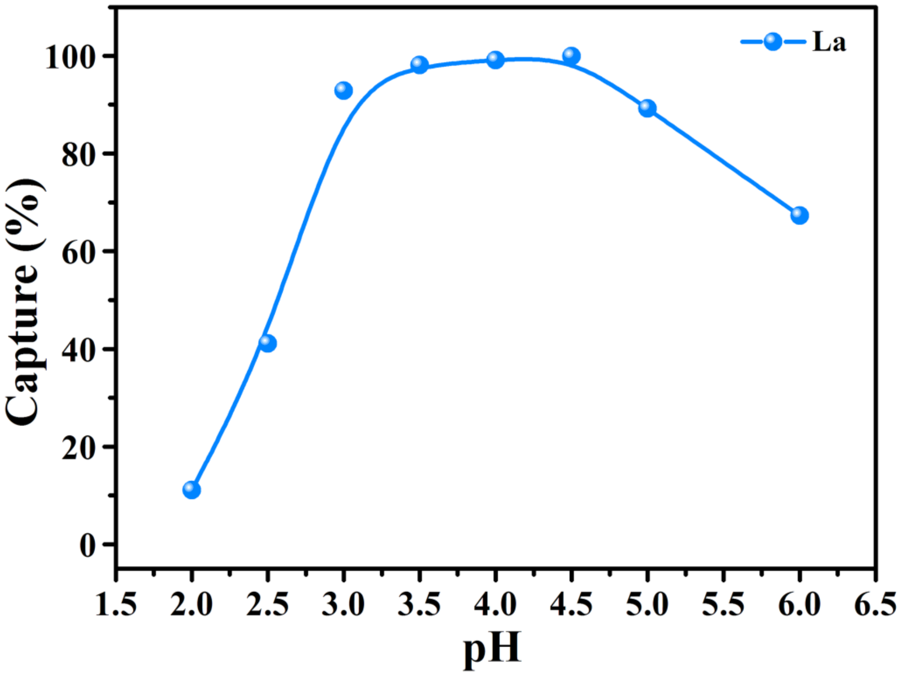

Supplementary Fig. 7 | Results of pH-dependent capture of La ions.

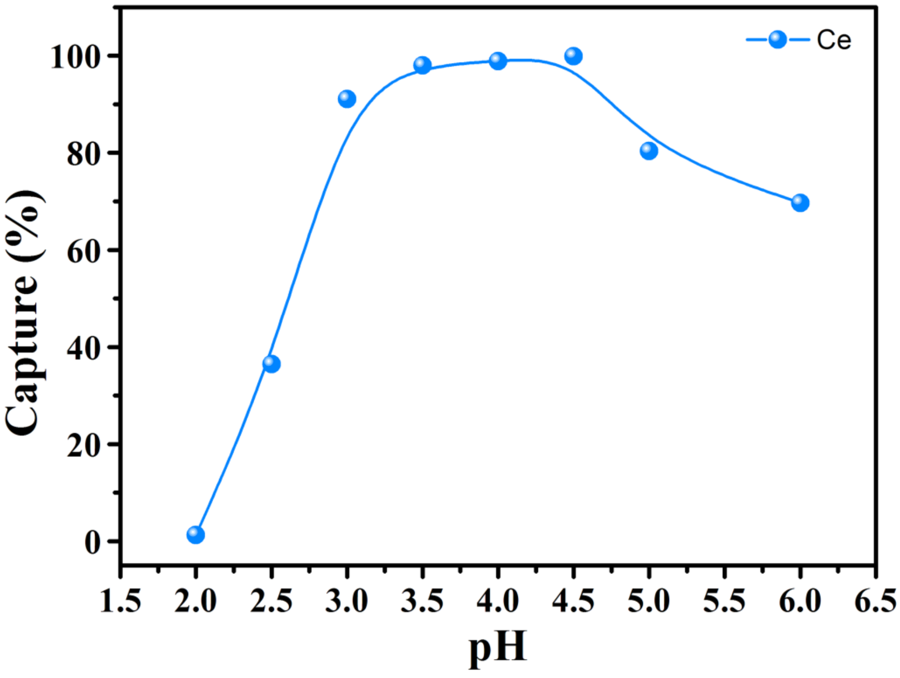

Supplementary Fig. 8 | Results of pH-dependent capture of Ce ions.

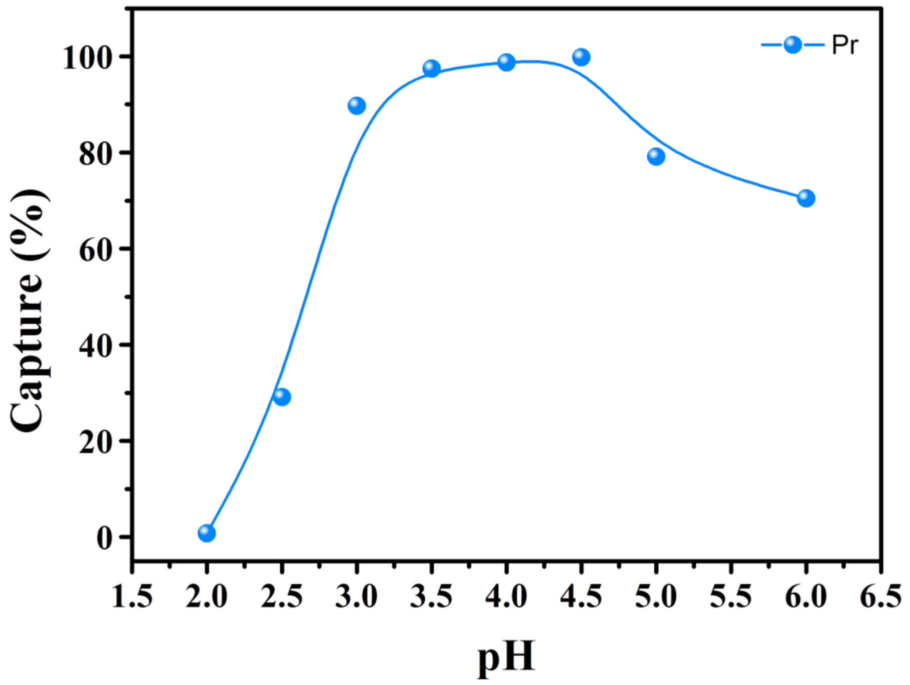

Supplementary Fig. 9 | Results of pH-dependent capture of Pr ions.

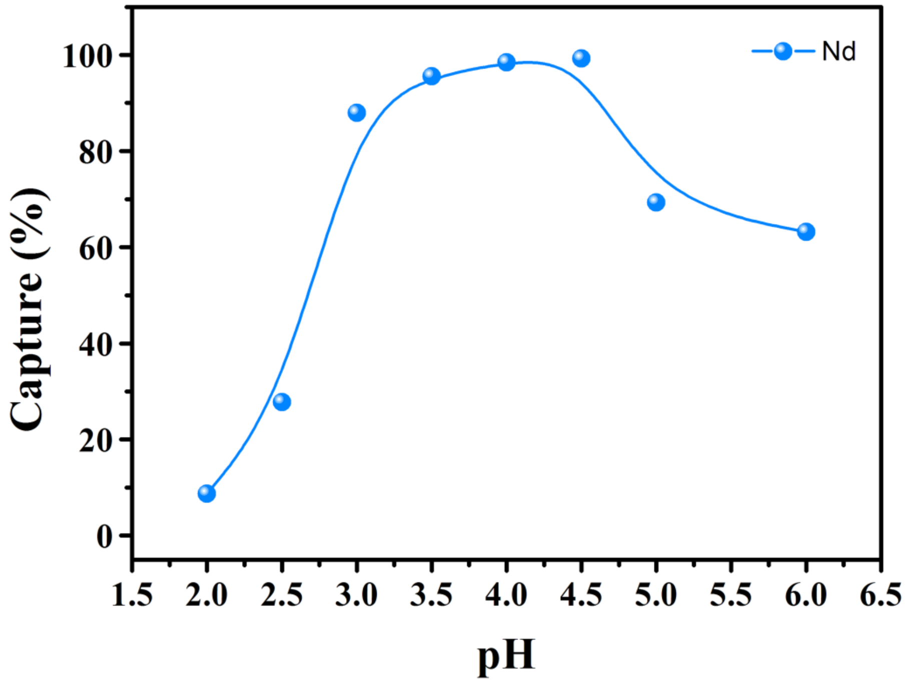

Supplementary Fig. 10 | Results of pH-dependent capture of Nd ions.

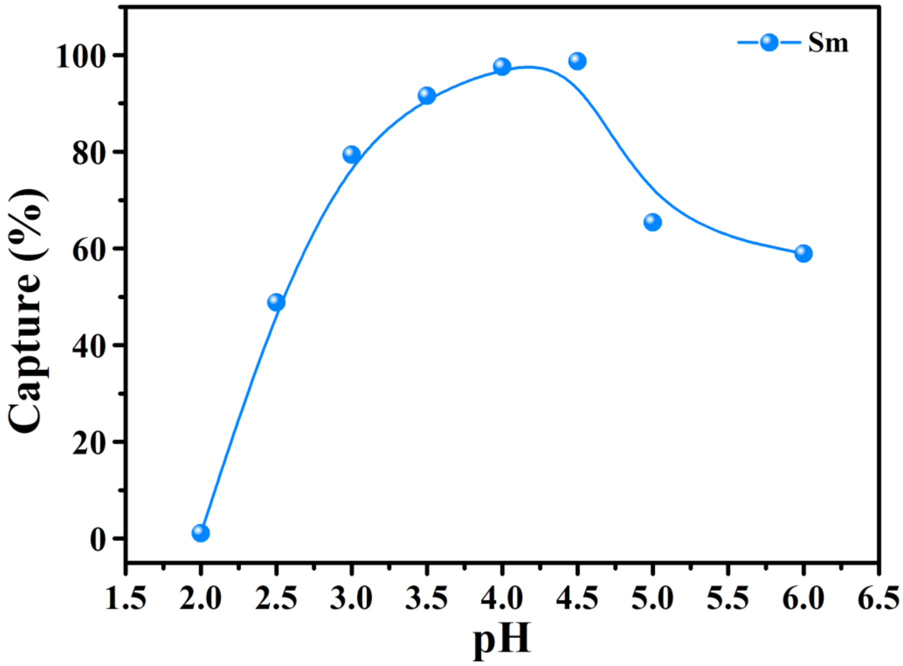

Supplementary Fig. 11 | Results of pH-dependent capture of Sm ions.

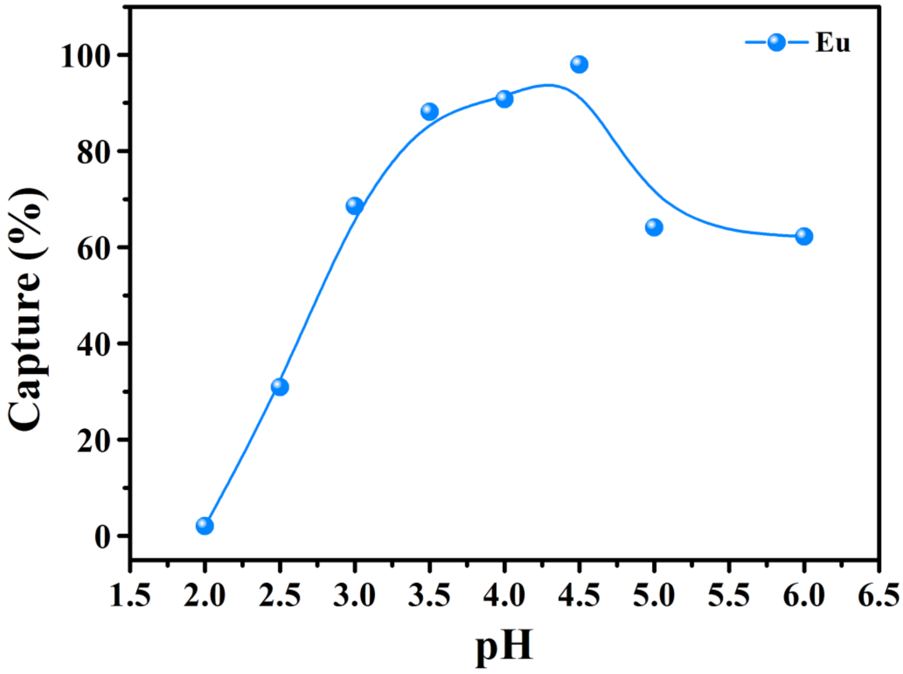

Supplementary Fig. 12 | Results of pH-dependent capture of Eu ions.

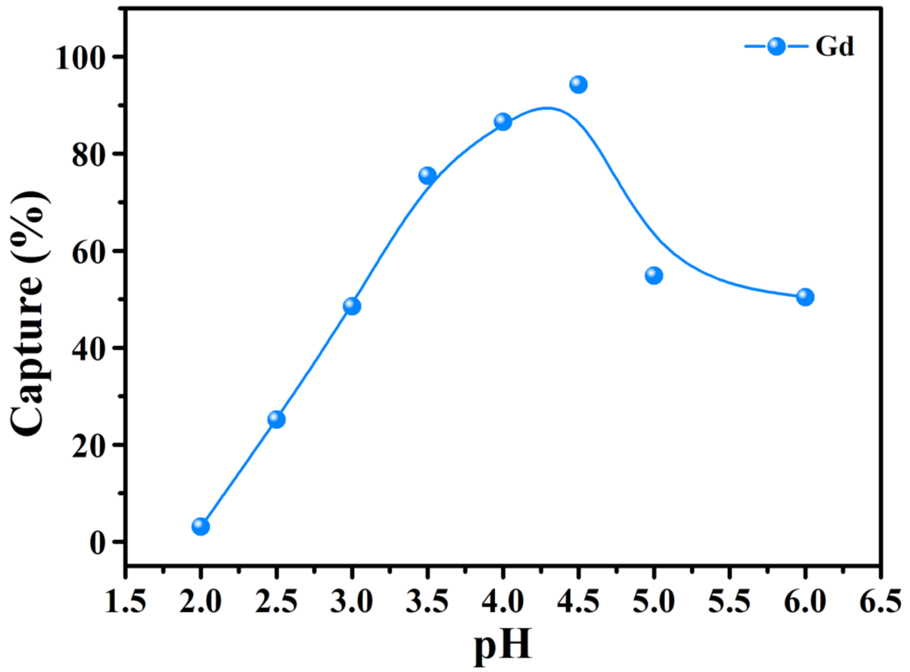

Supplementary Fig. 13 | Results of pH-dependent capture of Gd ions.

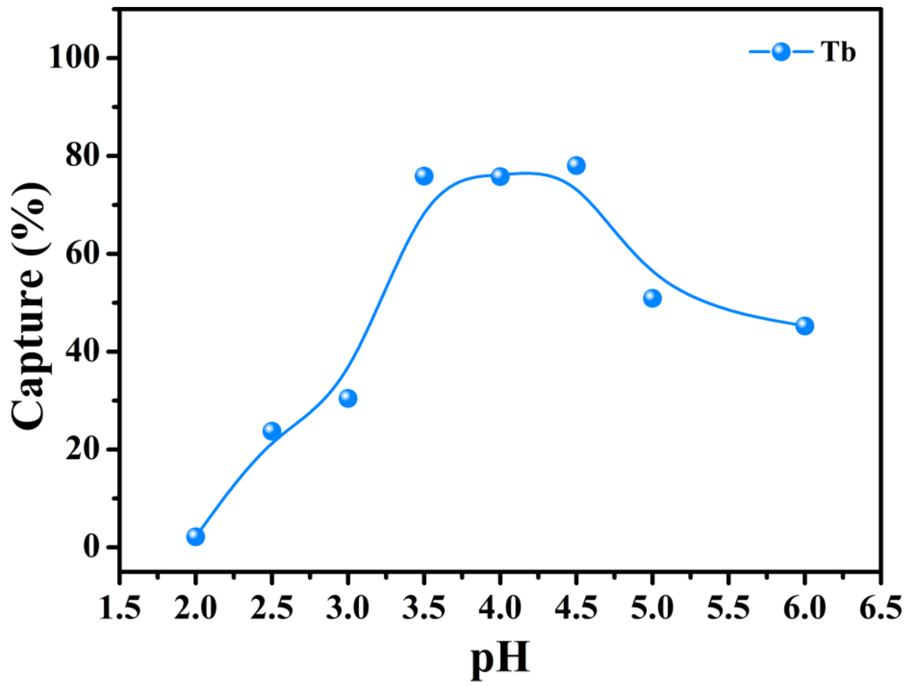

Supplementary Fig. 14 | Results of pH-dependent capture of Tb ions.

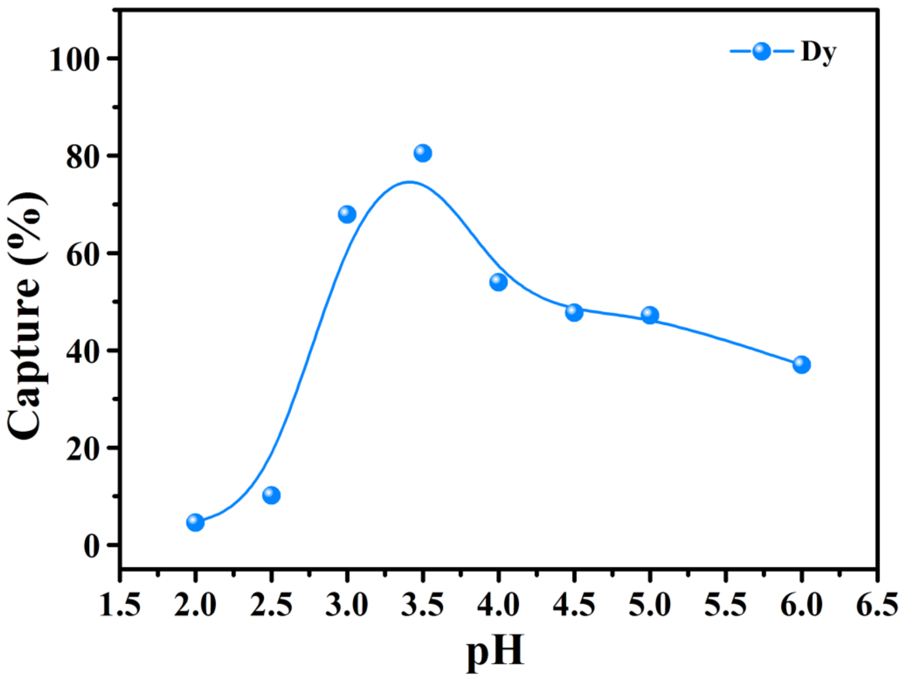

Supplementary Fig. 15 | Results of pH-dependent capture of Dy ions.

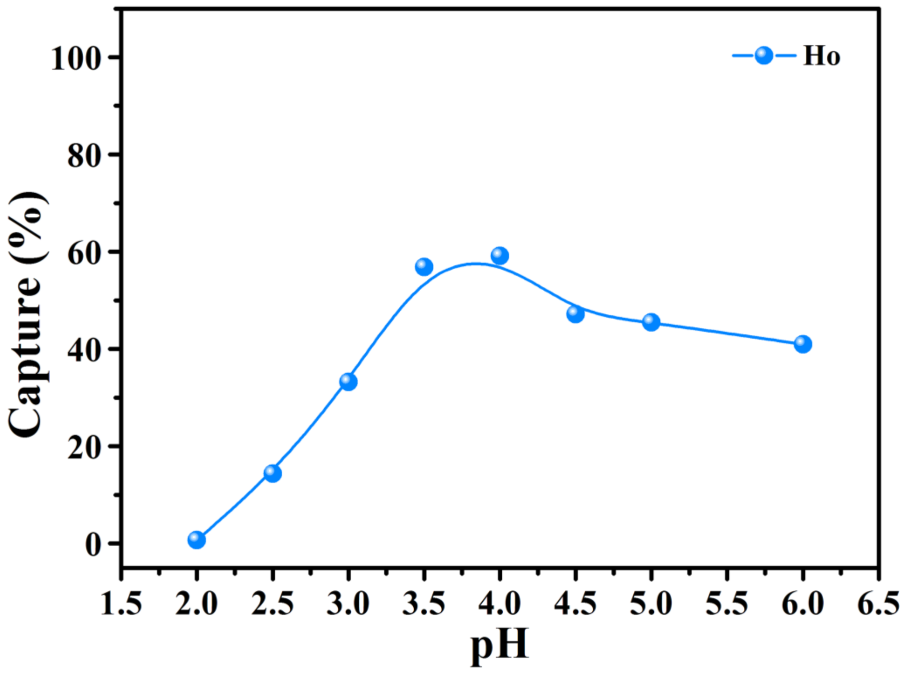

Supplementary Fig. 16 | Results of pH-dependent capture of Ho ions.

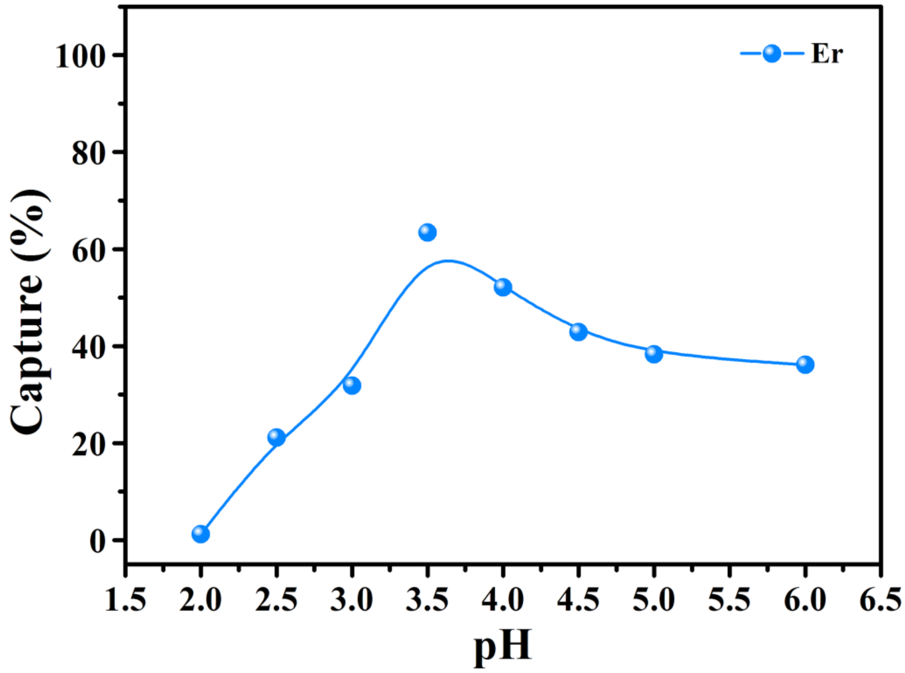

Supplementary Fig. 17 | Results of pH-dependent capture of Er ions.

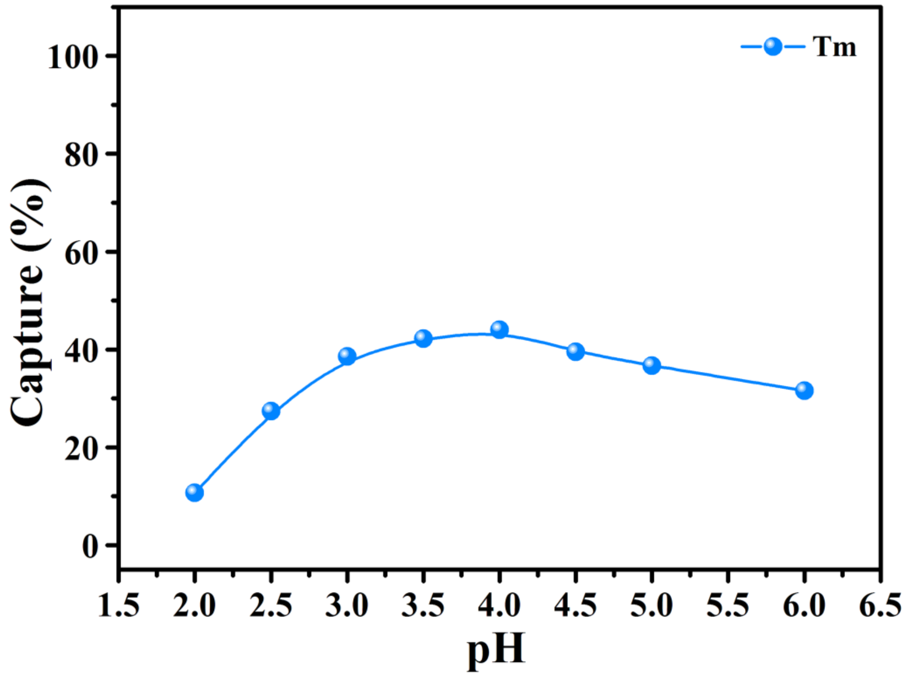

Supplementary Fig. 18 | Results of pH-dependent capture of Tm ions.

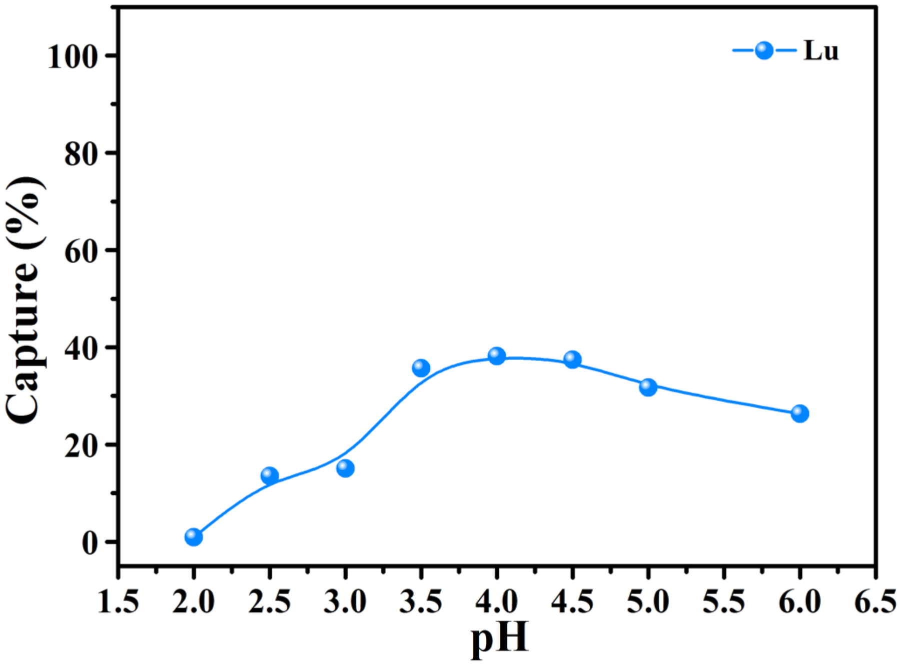

Supplementary Fig. 19 | Results of pH-dependent capture of Lu ions.

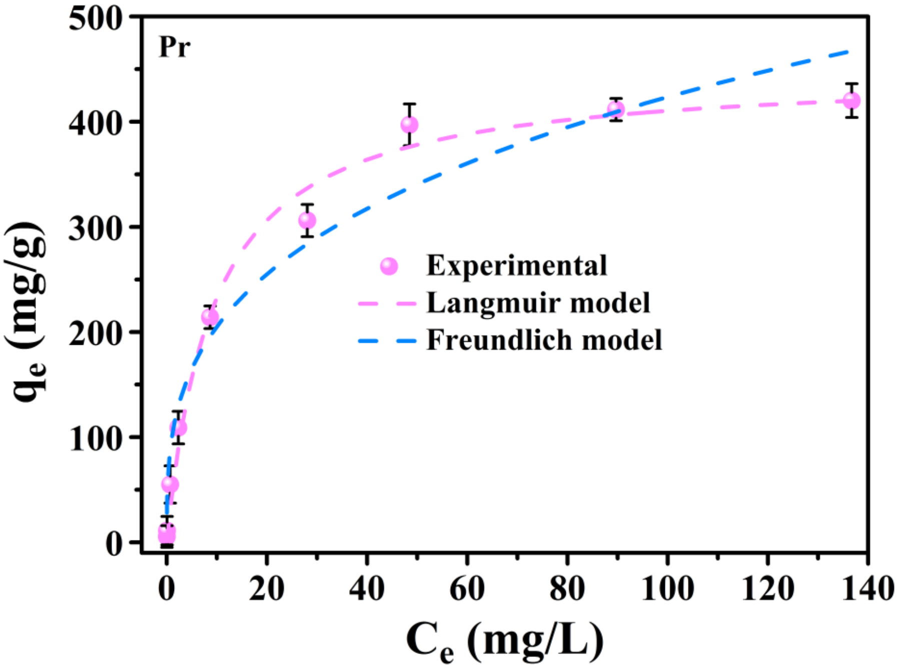

Supplementary Fig. 20 | Equilibrium data for Pr^{3+} adsorption at NCU-1 and fitted with the Langmuir and Freundlich isotherm models. Error bars represent S.D. n=3 independent experiments.

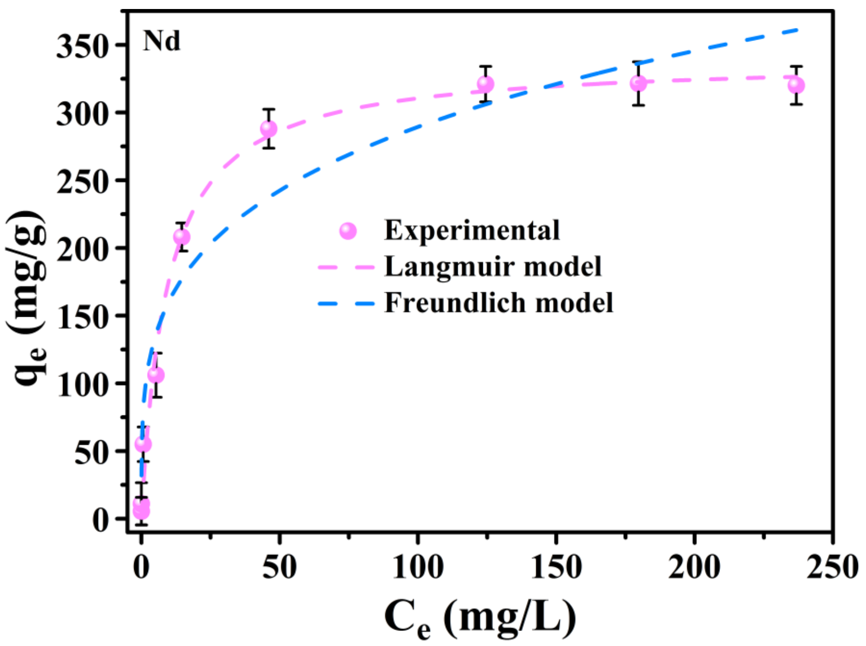

Supplementary Fig. 21 | Equilibrium data for Nd^{3+} adsorption at NCU-1 and fitted with the Langmuir and Freundlich isotherm models. Error bars represent S.D. n=3 independent experiments.

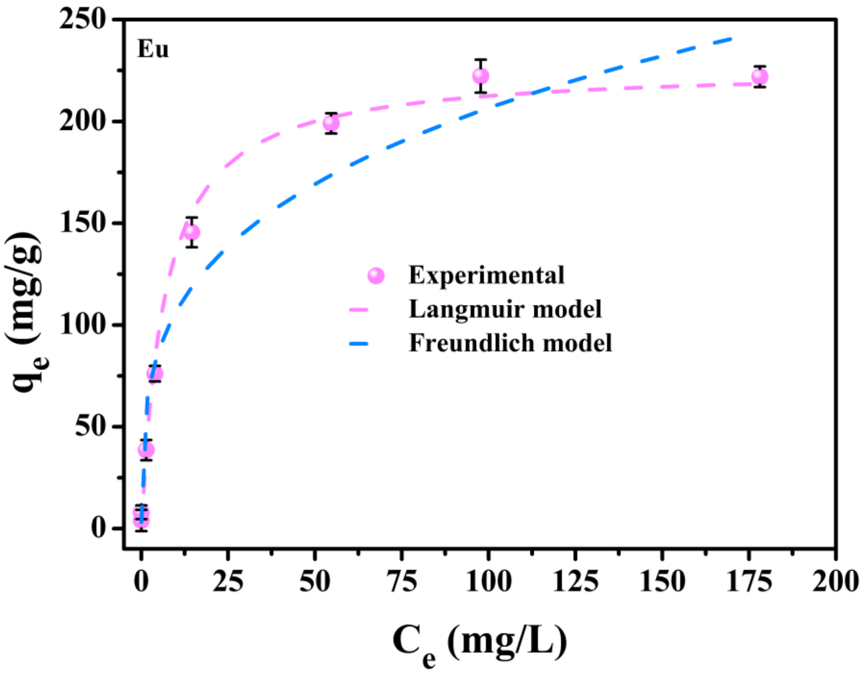

Supplementary Fig. 22 | Equilibrium data for Eu^{3+} adsorption at NCU-1 and fitted with the Langmuir and Freundlich isotherm models. Error bars represent S.D. n=3 independent experiments.

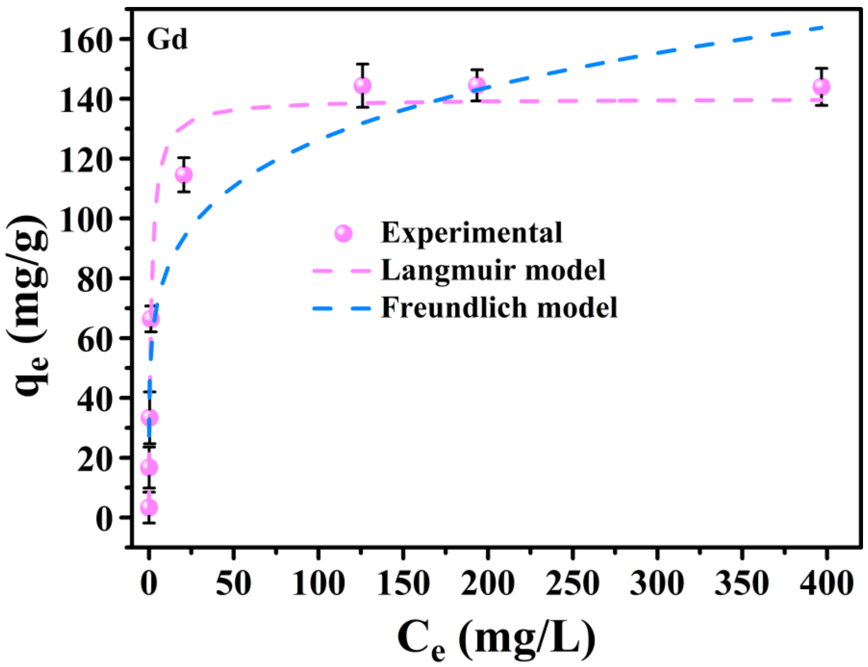

Supplementary Fig. 23 | Equilibrium data for Gd^{3+} adsorption at NCU-1 and fitted with the Langmuir and Freundlich isotherm models. Error bars represent S.D. n=3 independent experiments.

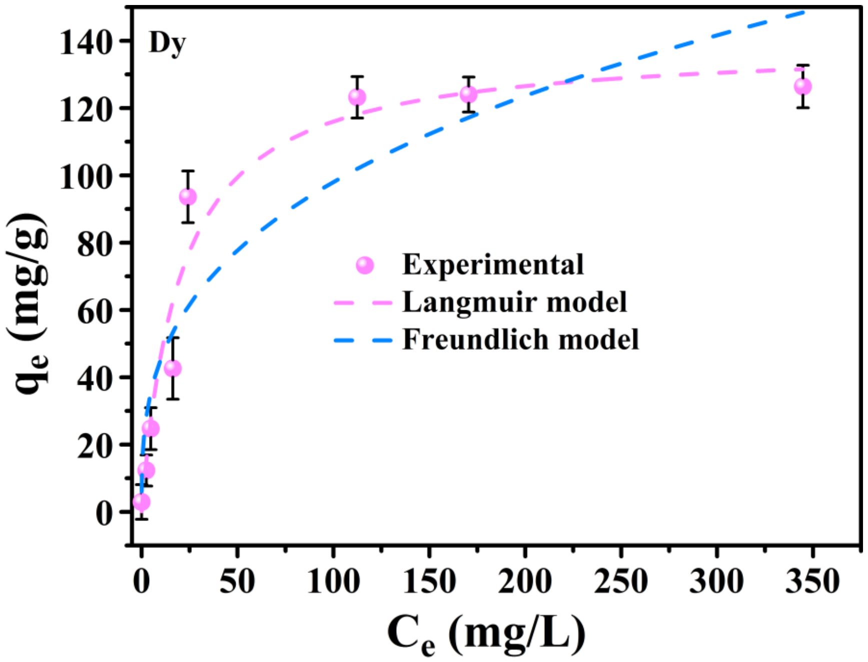

Supplementary Fig. 24 | Equilibrium data for Dy^{3+} adsorption at NCU-1 and fitted with the Langmuir and Freundlich isotherm models. Error bars represent S.D. n=3 independent experiments.

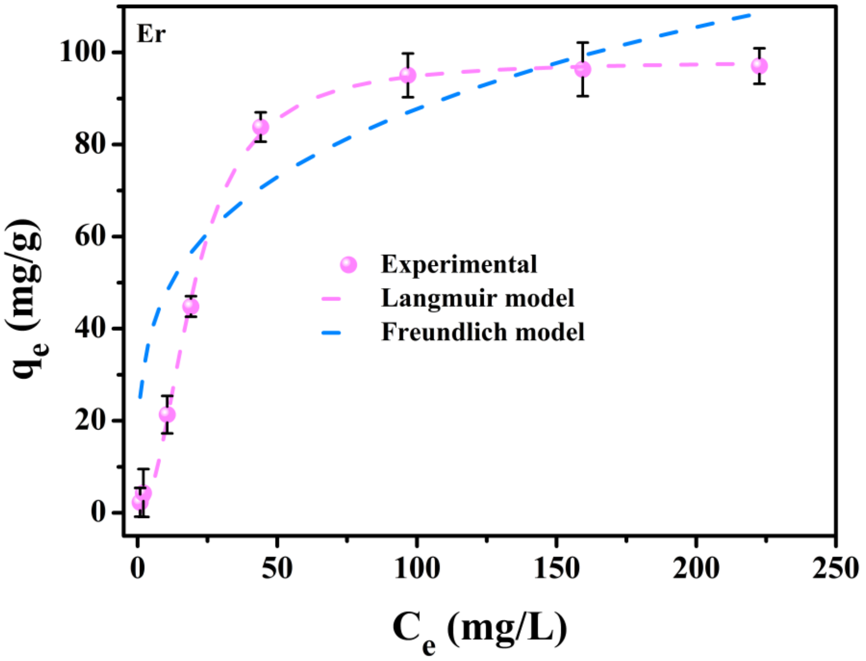

Supplementary Fig. 25 | Equilibrium data for Er^{3+} adsorption at NCU-1 and fitted with the Langmuir and Freundlich isotherm models. Error bars represent S.D. n=3 independent experiments.

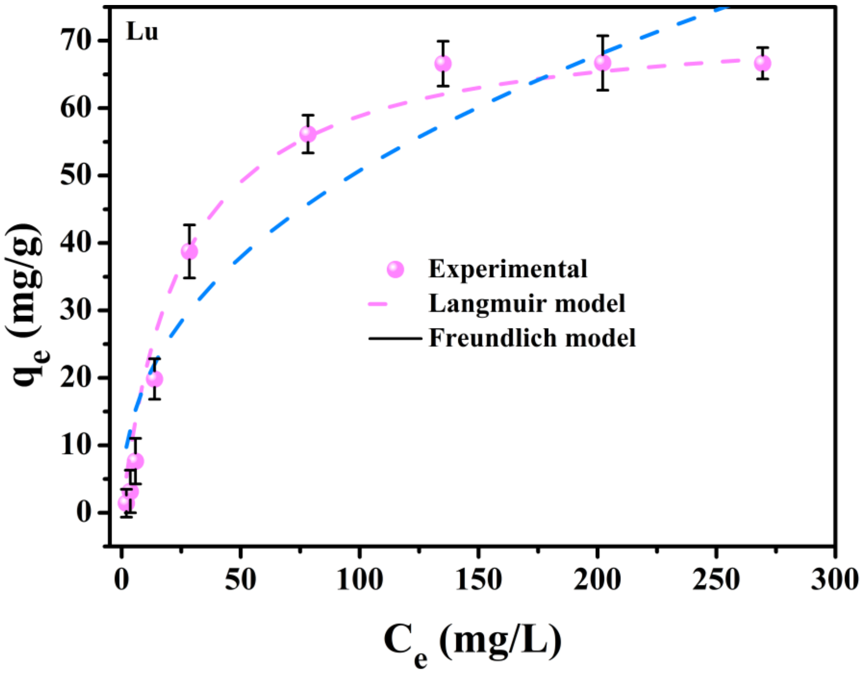

Supplementary Fig. 26 | Equilibrium data for Lu^{3+} adsorption at NCU-1 and fitted with the Langmuir and Freundlich isotherm models. Error bars represent S.D. n=3 independent experiments.

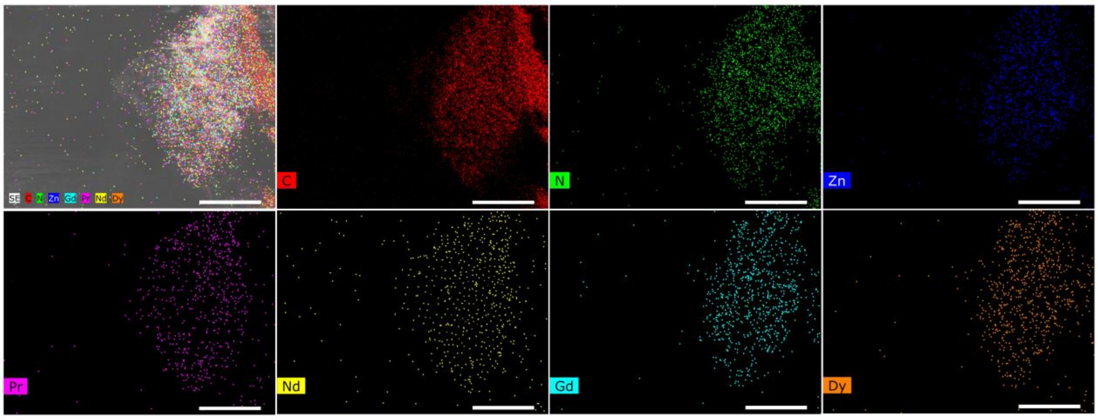

Supplementary Fig. 27 | EDS images of NCU-1 after adsorption REE (Pr^{3+}, Nd^{3+}, Gd^{3+}, Dy^{3+}) ions. Scale bars, 50 μm.

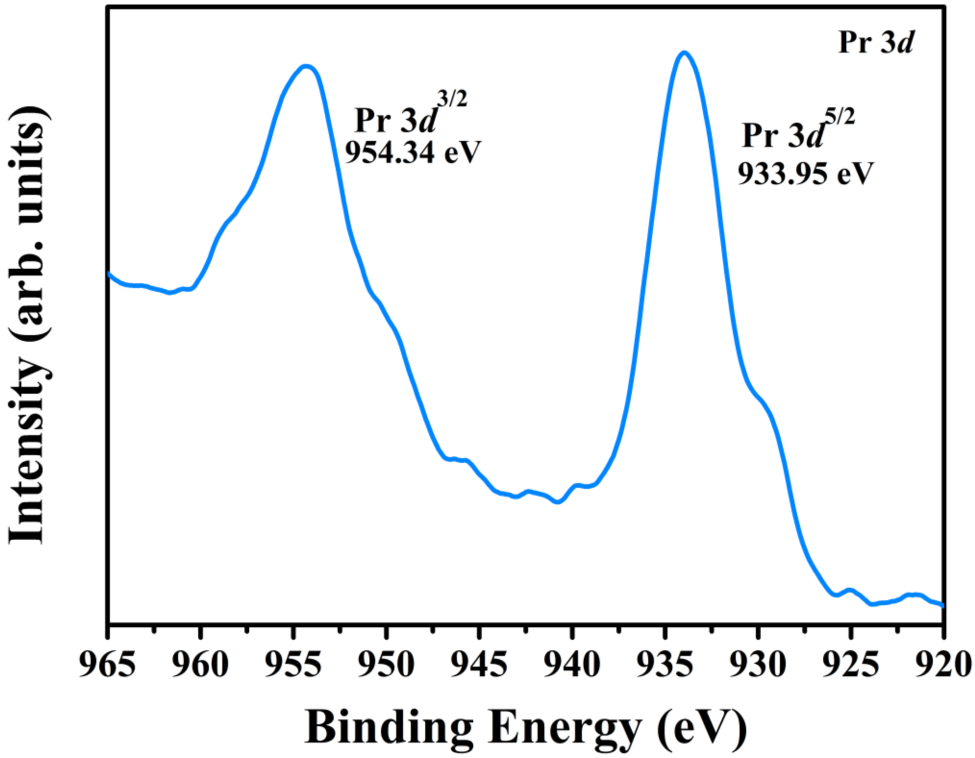

Supplementary Fig. 28 | XPS spectra of Pr 3*d*.

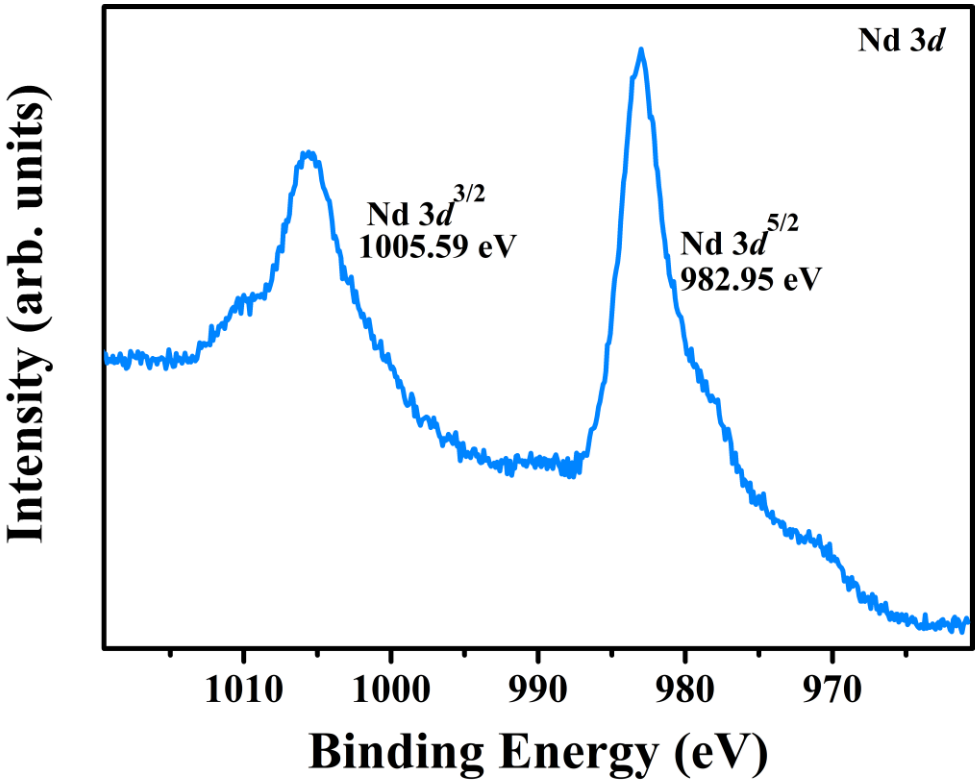

Supplementary Fig. 29 | XPS spectra of Nd 3*d*.

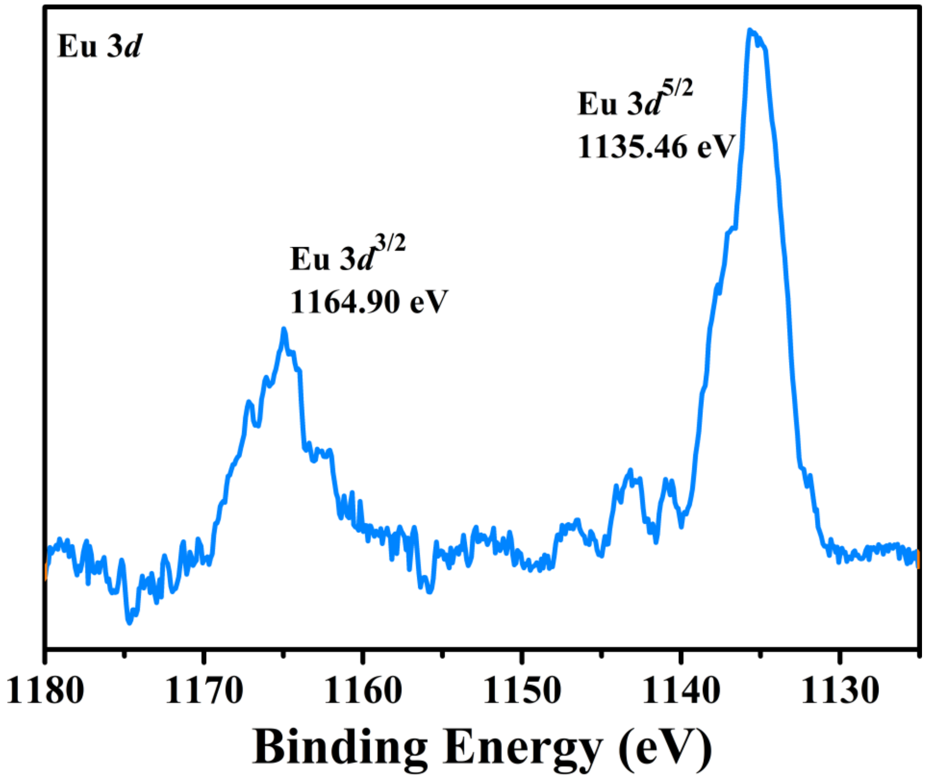

Supplementary Fig. 30 | XPS spectra of Eu 3*d*.

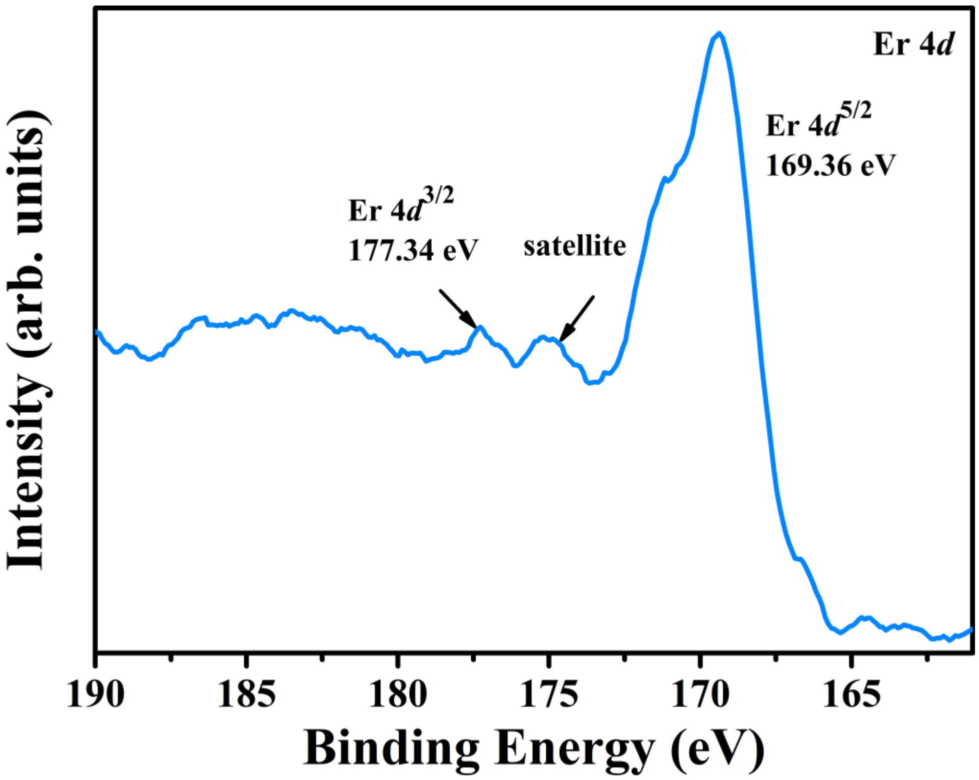

Supplementary Fig. 31 | XPS spectra of Er 4*d*.

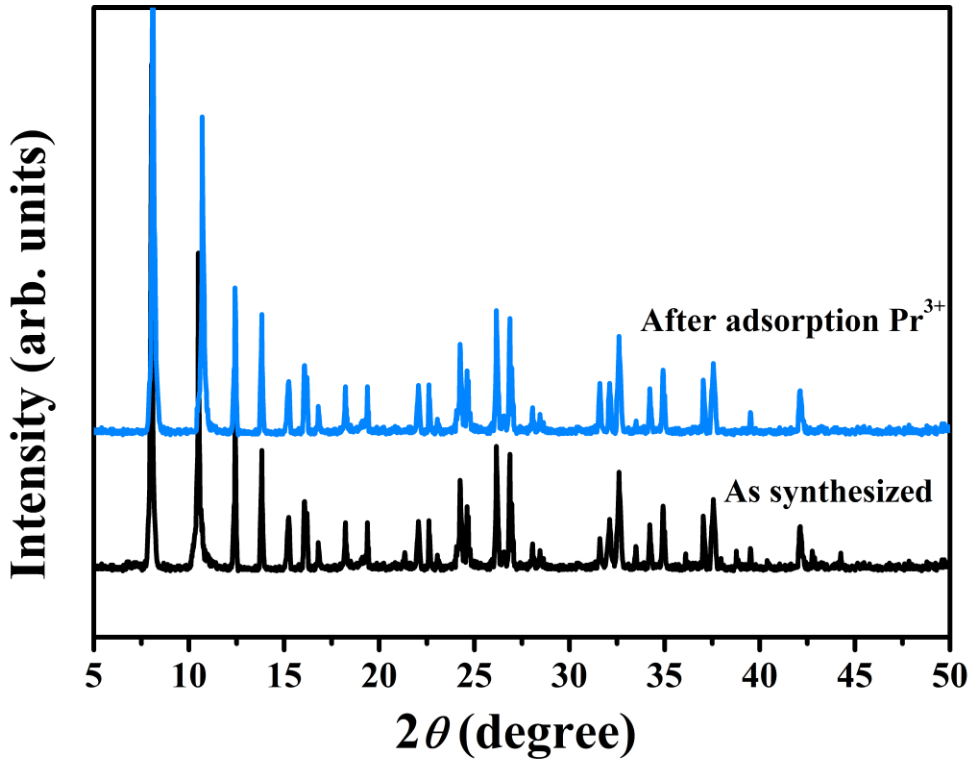

Supplementary Fig. 32 | PXRD patterns after capturing Pr^{3+}.

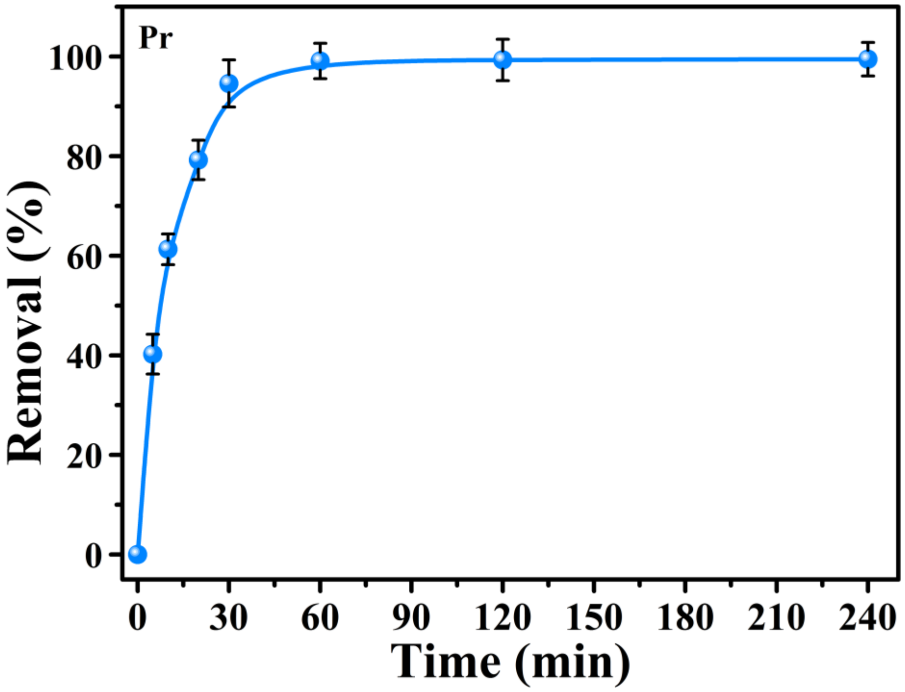

Supplementary Fig. 33 | The kinetics curve of Pr^{3+} adsorption. Error bars represent S.D. n=3 independent experiments.

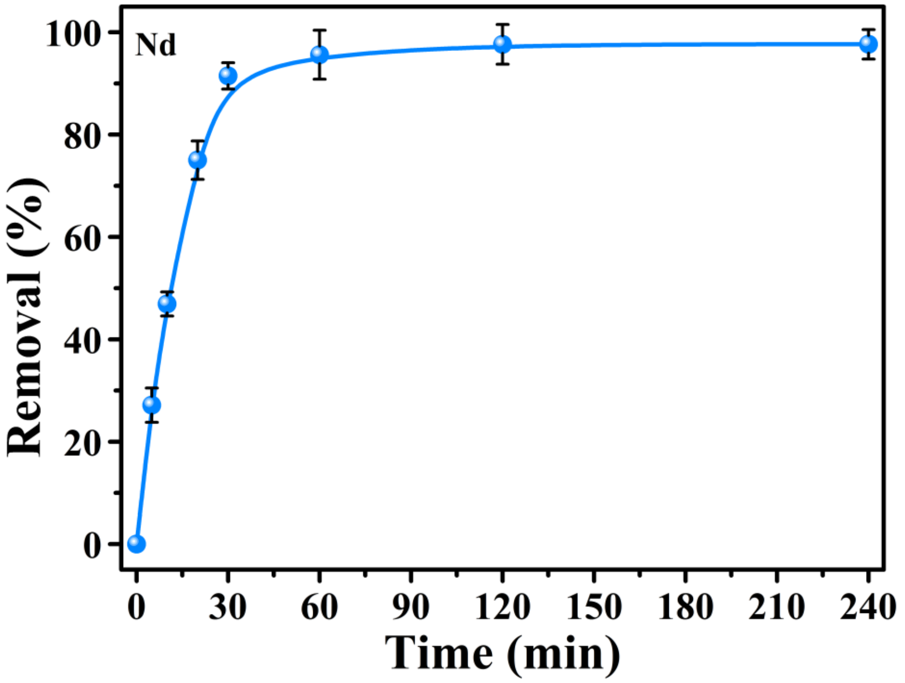

Supplementary Fig. 34 | The kinetics curve of Nd^{3+} adsorption. Error bars represent S.D. n=3 independent experiments.

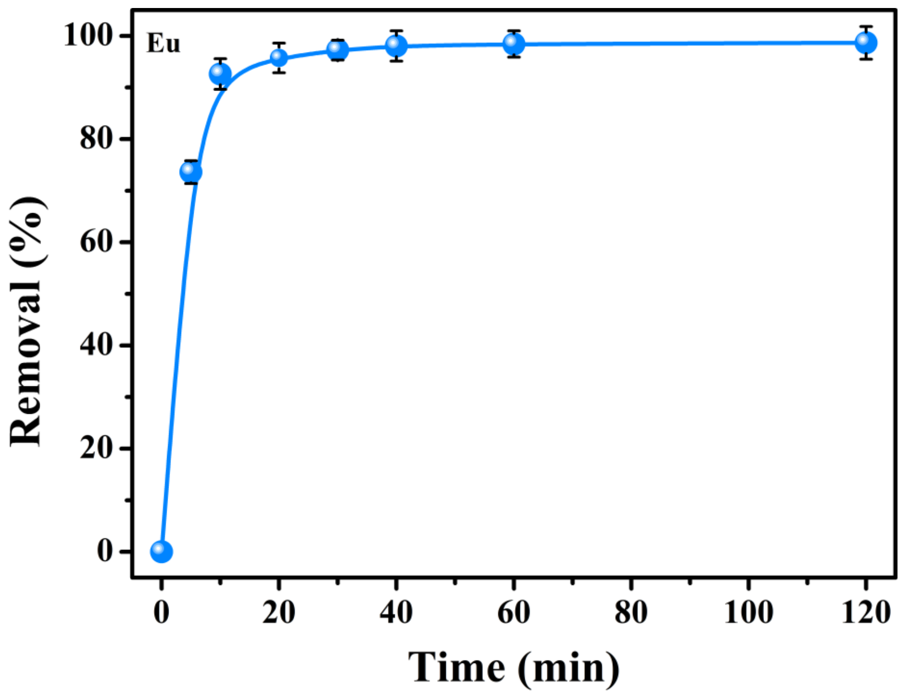

Supplementary Fig. 35 | The kinetics curve of Eu^{3+} adsorption. Error bars represent S.D. n=3 independent experiments.

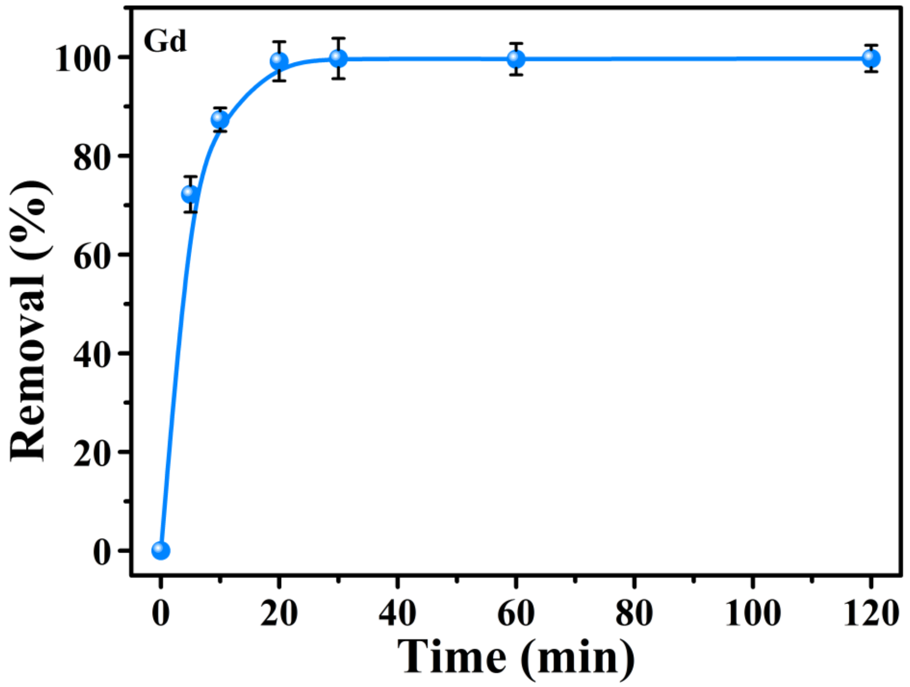

Supplementary Fig. 36 | The kinetics curve of Gd^{3+} adsorption. Error bars represent S.D. n=3 independent experiments.

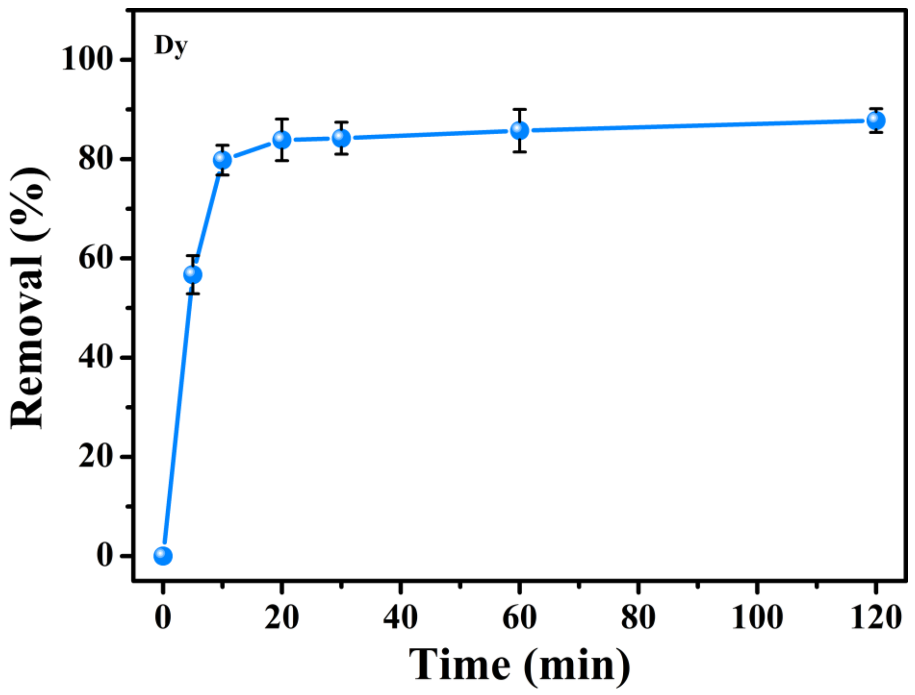

Supplementary Fig. 37 | The kinetics curve of Dy^{3+} adsorption. Error bars represent S.D. n=3 independent experiments.

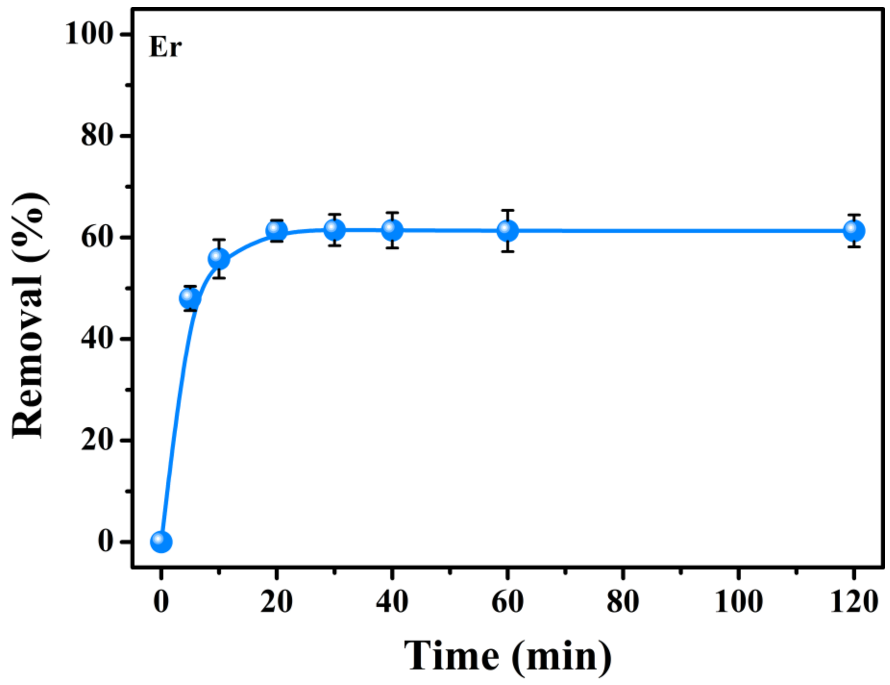

Supplementary Fig. 38 | The kinetics curve of Er^{3+} adsorption. Error bars represent S.D. n=3 independent experiments.

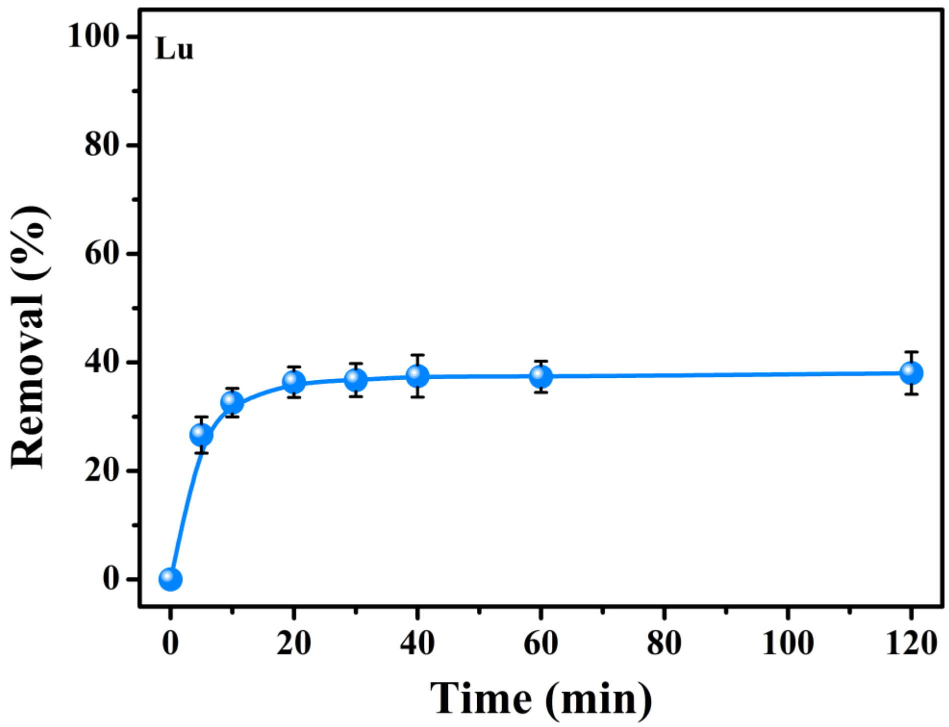

Supplementary Fig. 39 | The kinetics curve of Lu^{3+} adsorption. Error bars represent S.D. n=3 independent experiments.

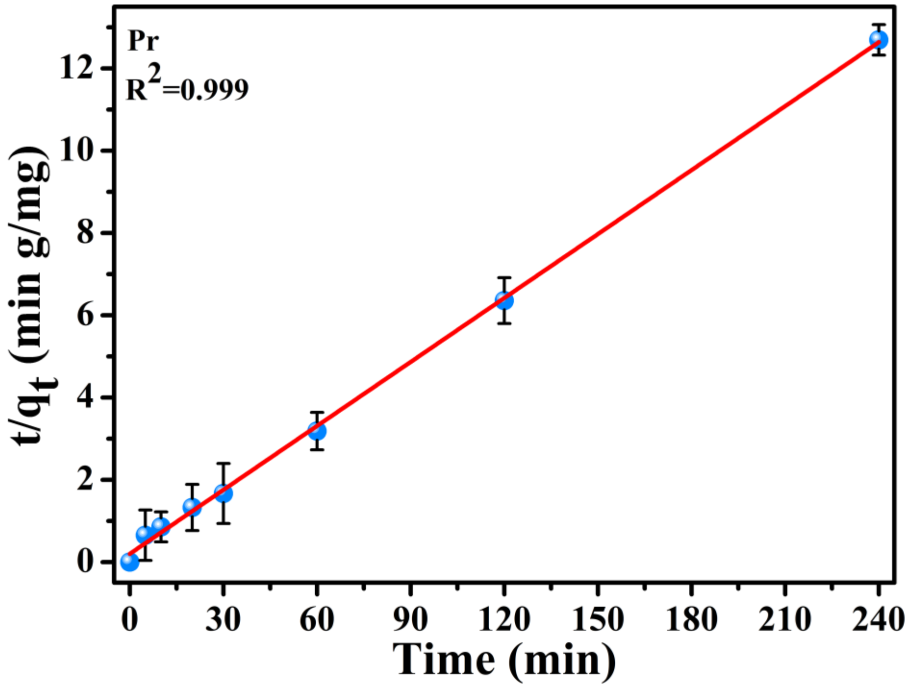

Supplementary Fig. 40 | The fitting curve based on the pseudo-second-order model of Pr^{3+}. Error bars represent S.D. n=3 independent experiments.

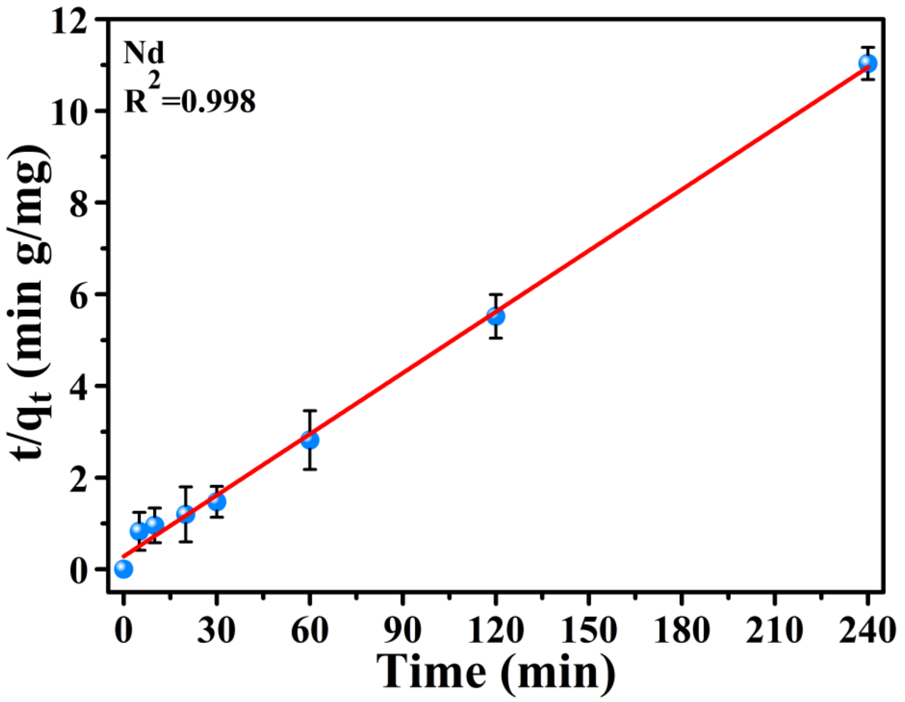

Supplementary Fig. 41 | The fitting curve based on the pseudo-second-order model of Nd^{3+}. Error bars represent S.D. n=3 independent experiments.

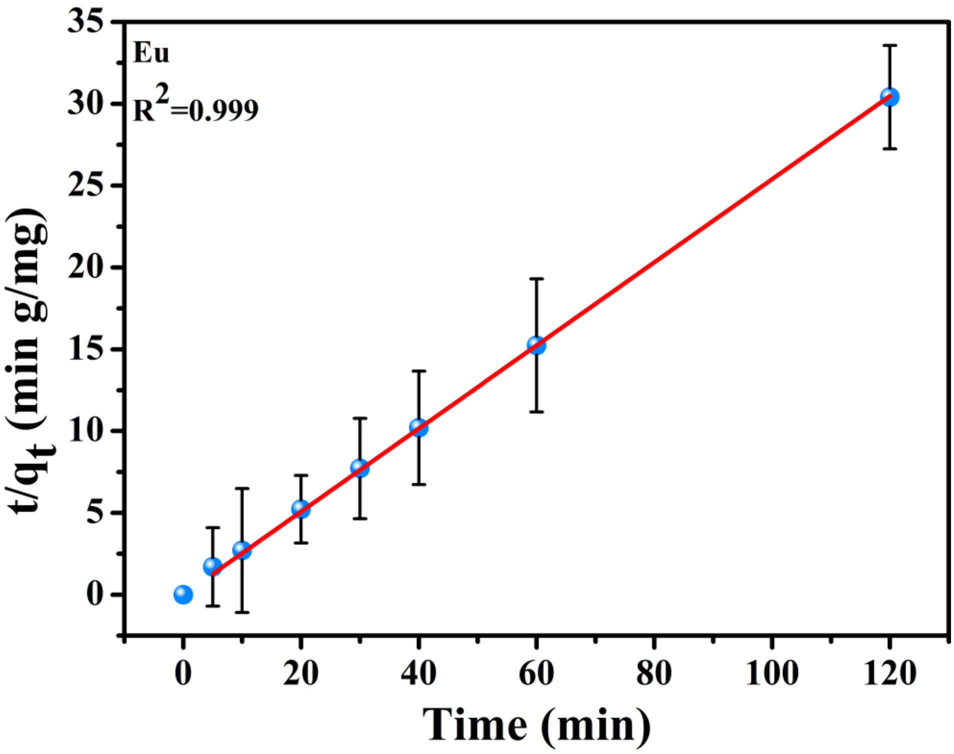

Supplementary Fig. 42 | The fitting curve based on the pseudo-second-order model of Eu^{3+}. Error bars represent S.D. n=3 independent experiments.

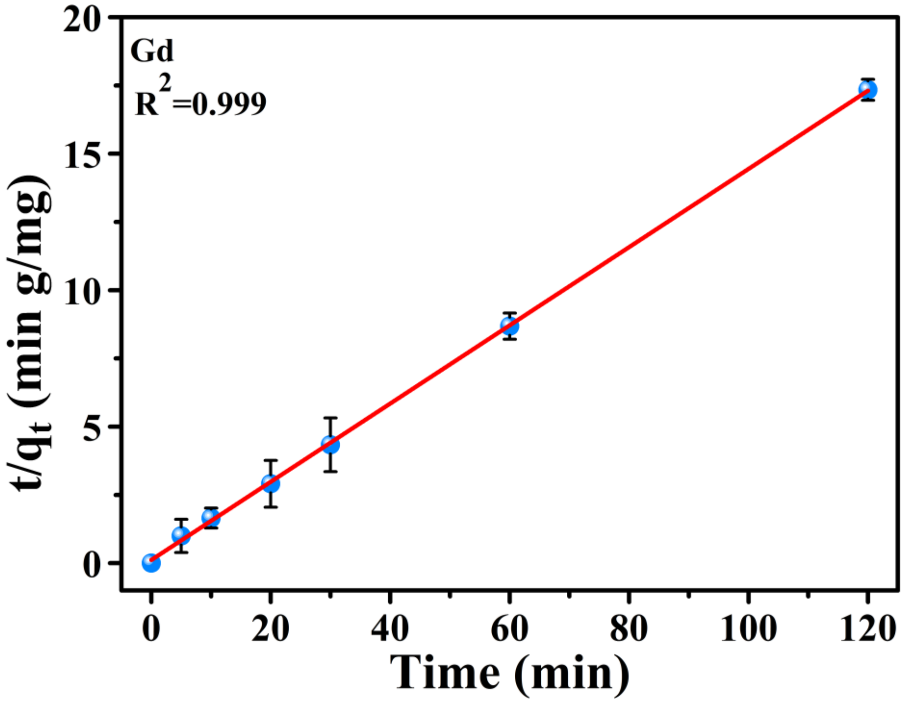

Supplementary Fig. 43 | The fitting curve based on the pseudo-second-order model of Gd^{3+}. Error bars represent S.D. n=3 independent experiments.

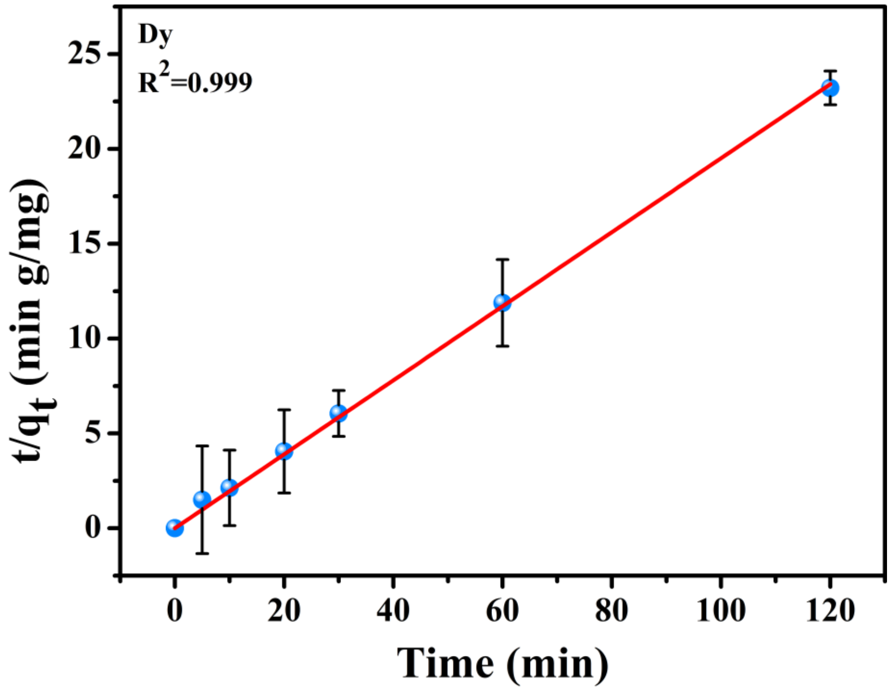

Supplementary Fig. 44 | The fitting curve based on the pseudo-second-order model of Dy^{3+}. Error bars represent S.D. n=3 independent experiments.

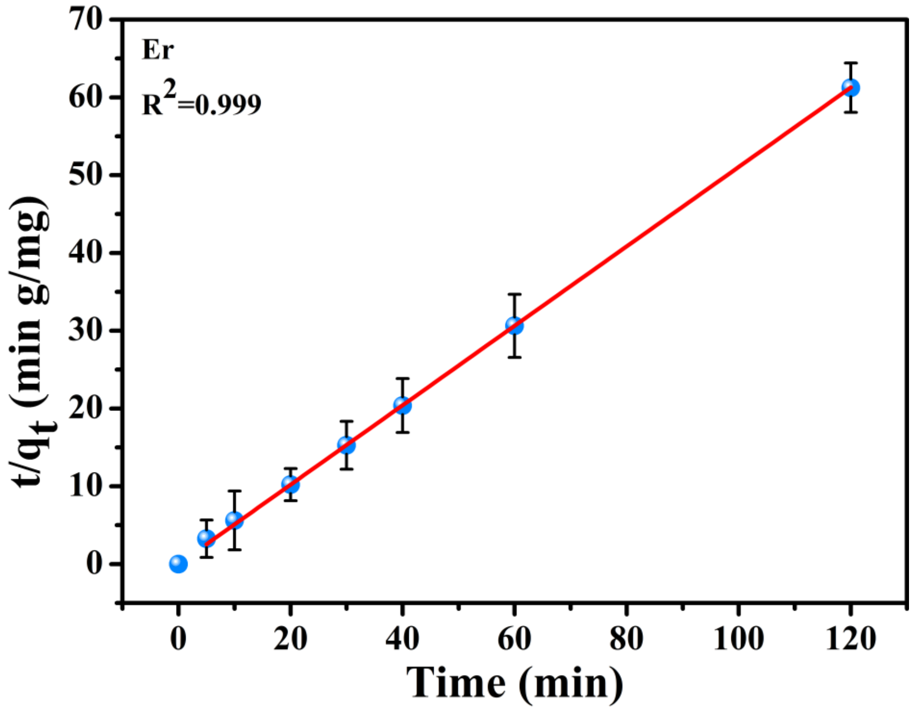

Supplementary Fig. 45 | The fitting curve based on the pseudo-second-order model of Er^{3+}. Error bars represent S.D. n=3 independent experiments.

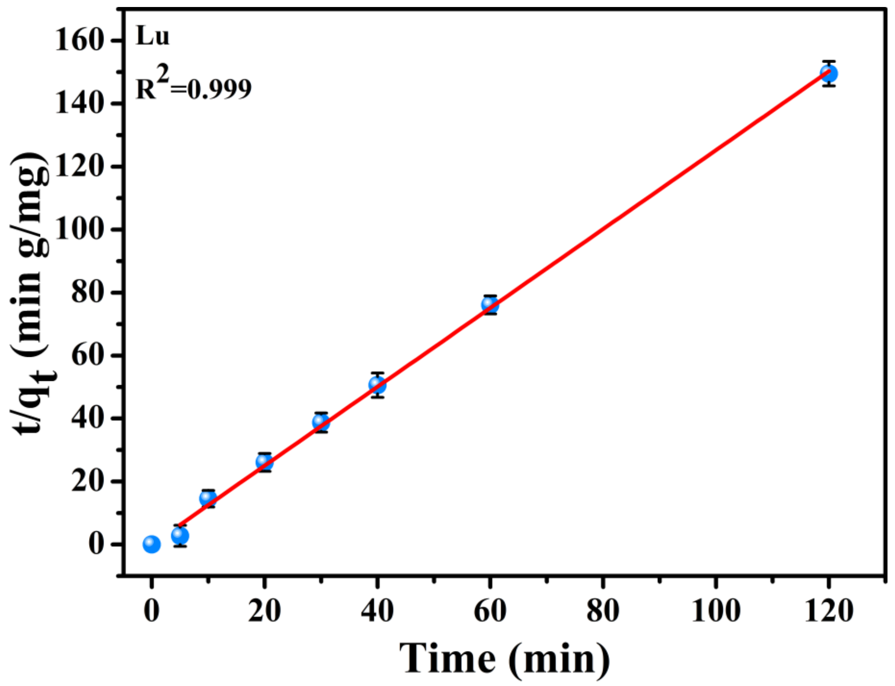

Supplementary Fig. 46 | The fitting curve based on the pseudo-second-order model of Lu^{3+}. Error bars represent S.D. n=3 independent experiments.

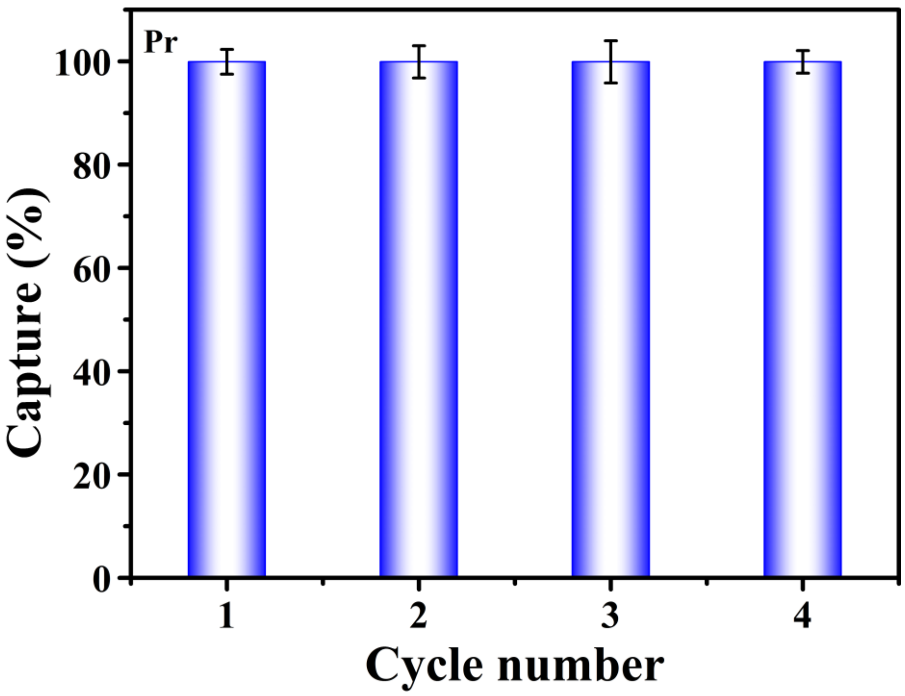

Supplementary Fig. 47 | Reusability of NCU-1 for capturing Pr^{3+}. Error bars represent S.D. n=3 independent experiments.

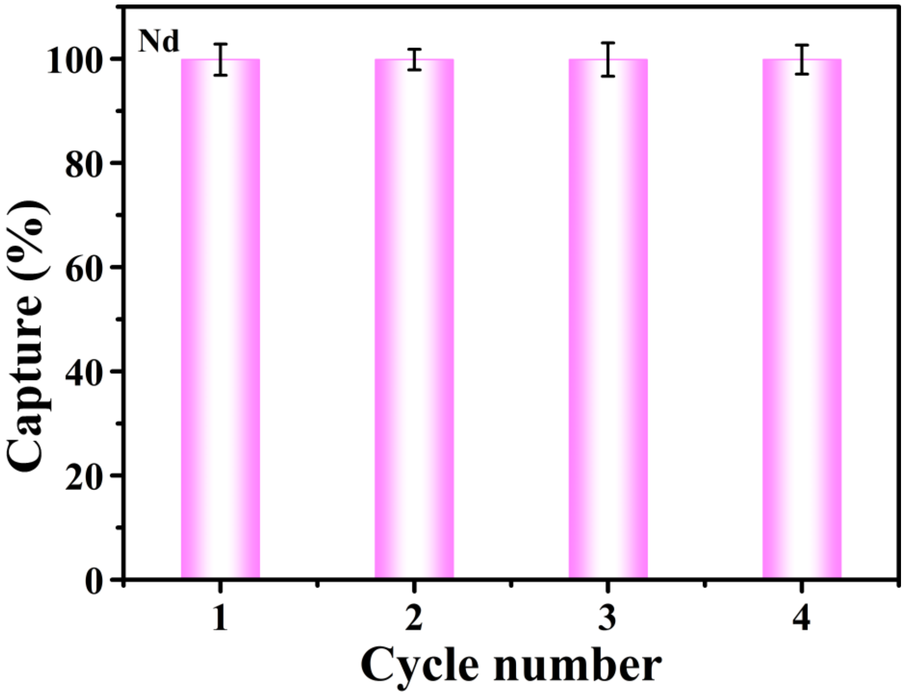

Supplementary Fig. 48 | Reusability of NCU-1 for capturing Nd^{3+}. Error bars represent S.D. n=3 independent experiments.

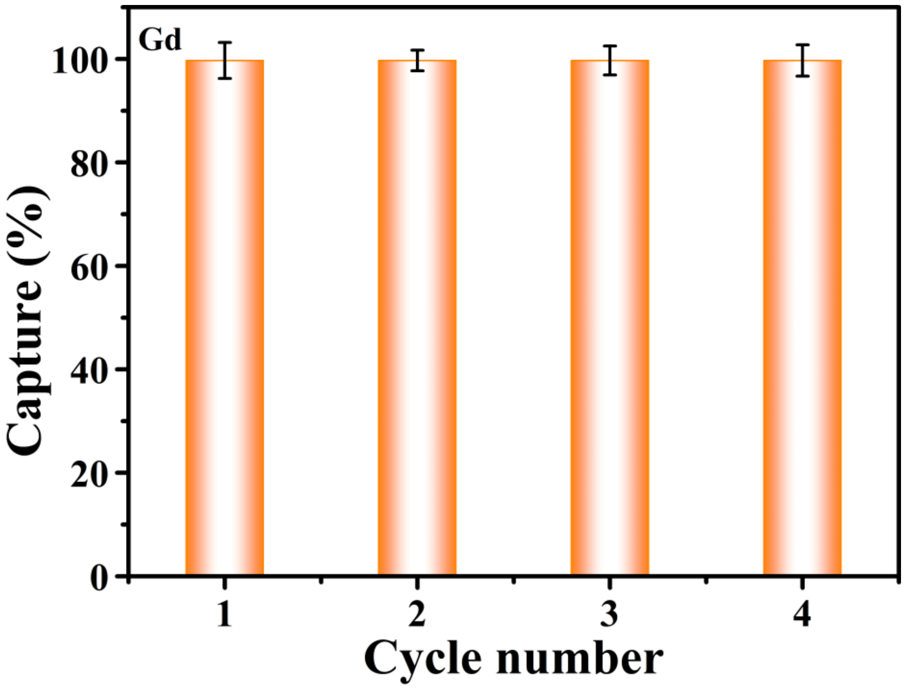

Supplementary Fig. 49 | Reusability of NCU-1 for capturing Gd^{3+}. Error bars represent S.D. n=3 independent experiments.

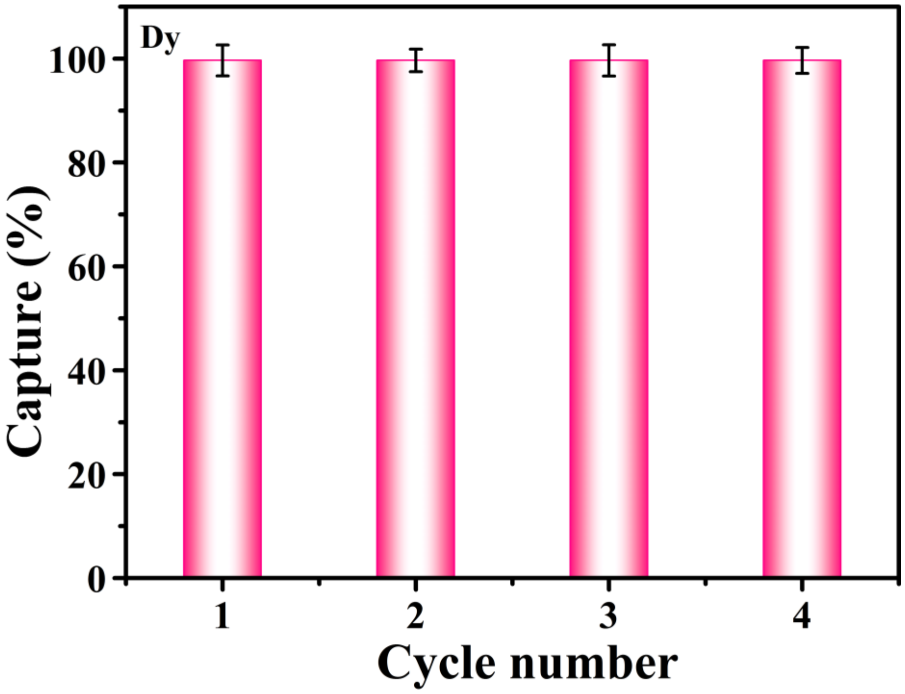

Supplementary Fig. 50 | Reusability of NCU-1 for capturing Dy^{3+}. Error bars represent S.D. n=3 independent experiments.

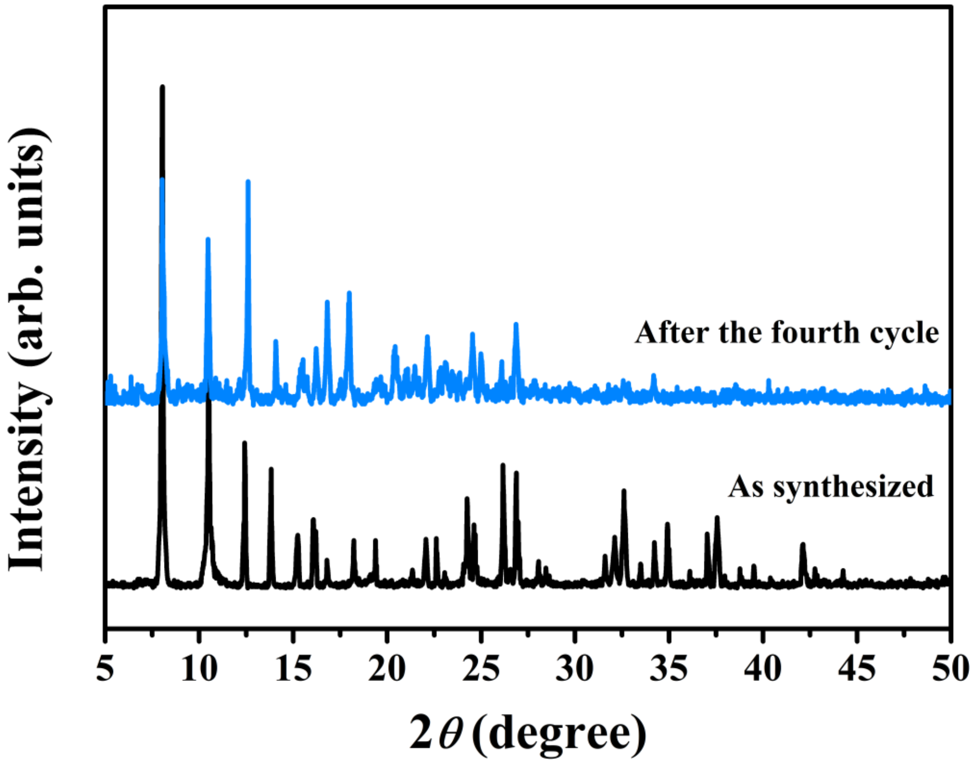

Supplementary Fig. 51 | PXRD patterns of NCU-1 after the fourth cycle.

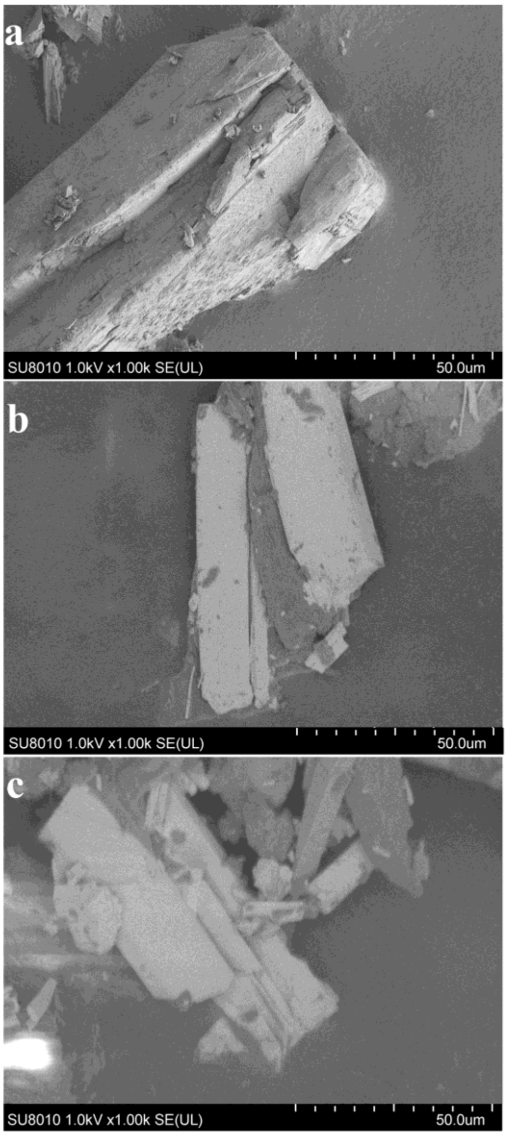

Supplementary Fig. 52 | ** SEM images of NCU-1 **a, after exposition to water solutions 7 days **b**, and after the fourth reuse of NCU-1 **c**.

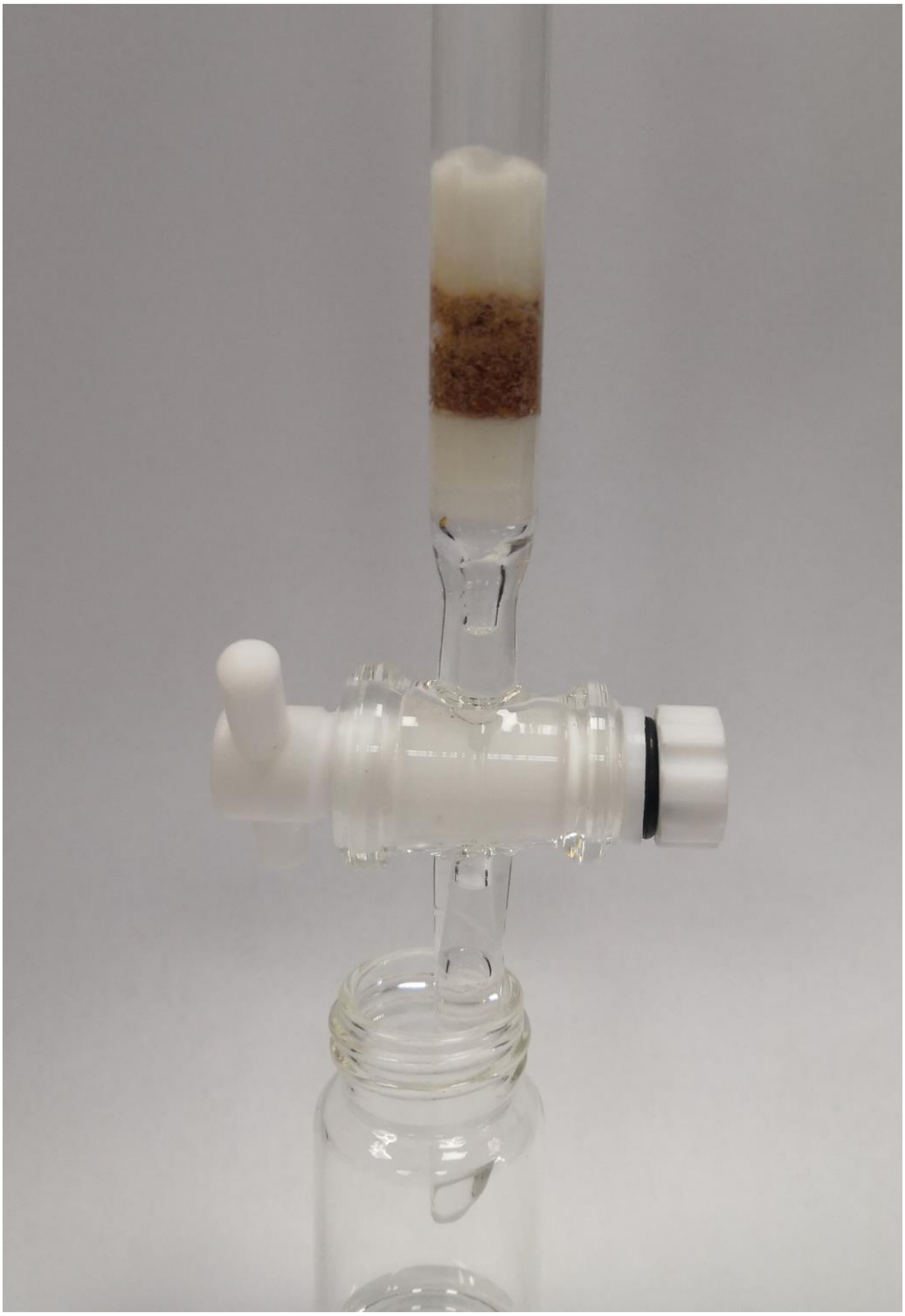

Supplementary Fig. 53 | The picture of the experimental breakthrough of mine tailing collected from Ganzhou city.

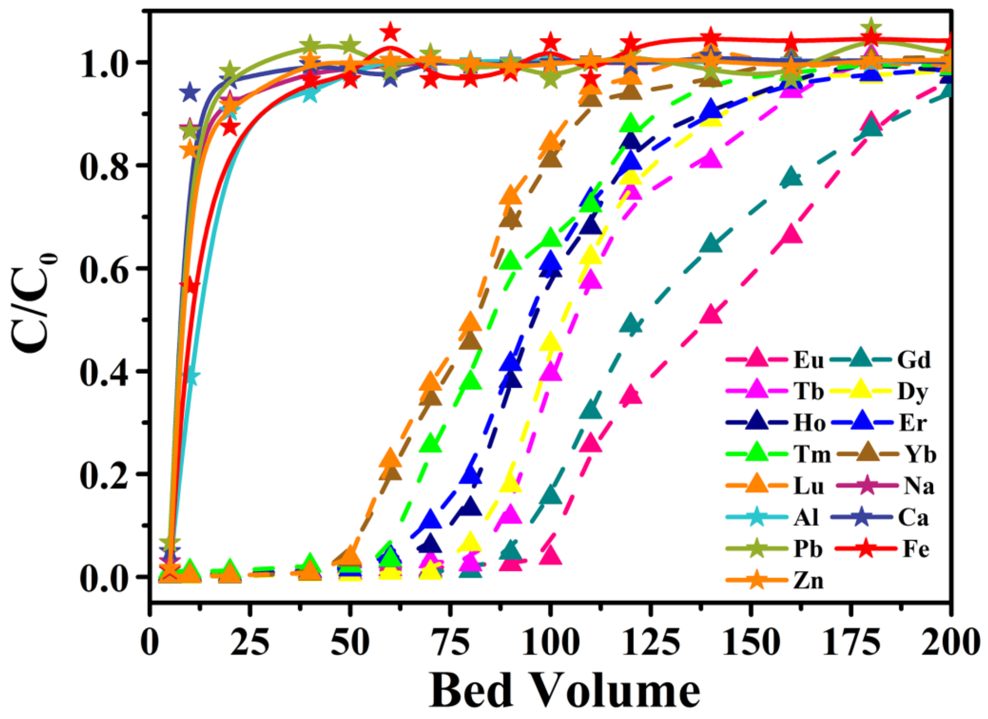

Supplementary Fig. 54 | Mine tailing breakthrough curves of interfering metal ions and heavy REE ions.

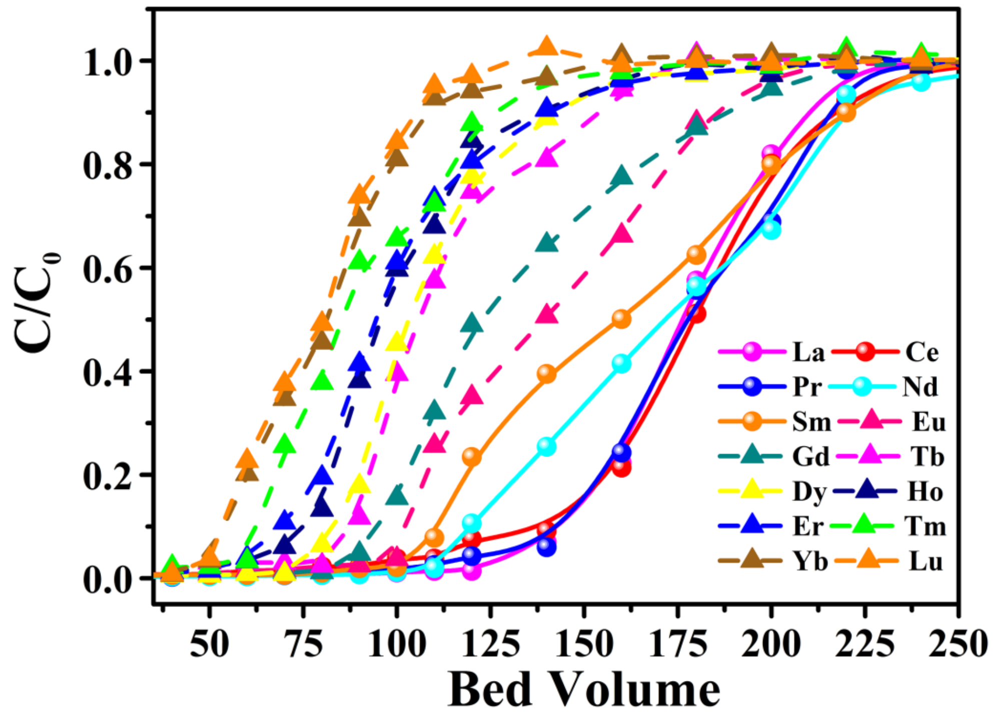

Supplementary Fig. 55 | Mine tailing breakthrough curves of heavy REE ions and light REE ions.

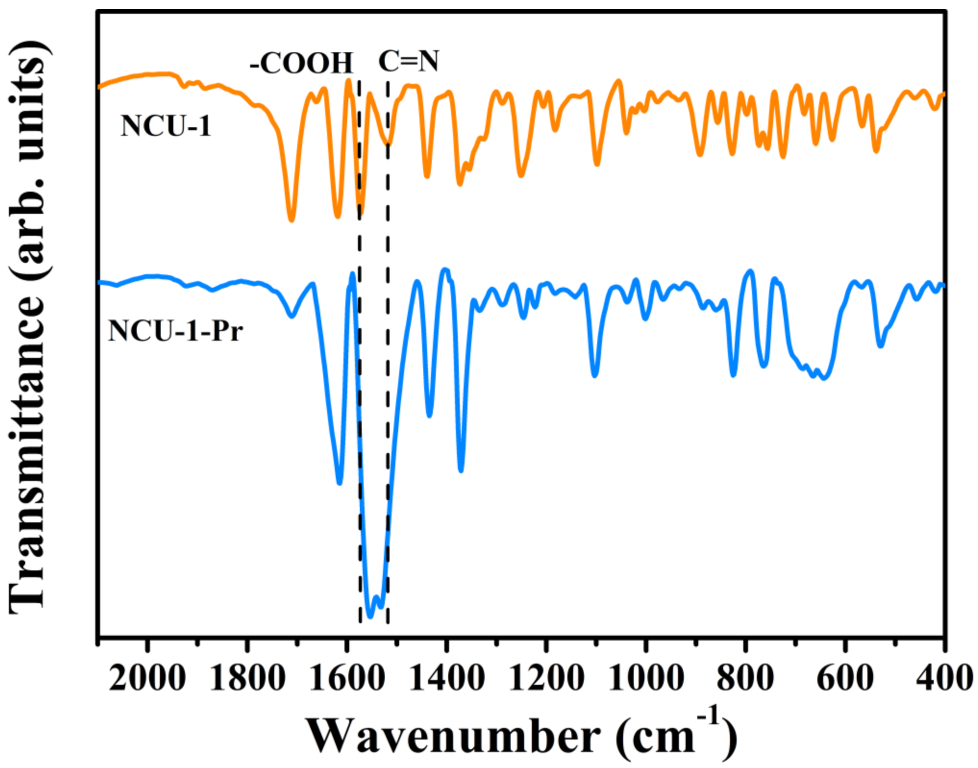

Supplementary Fig. 56 | FT-IR spectra of NCU-1 and NCU-1-Pr.

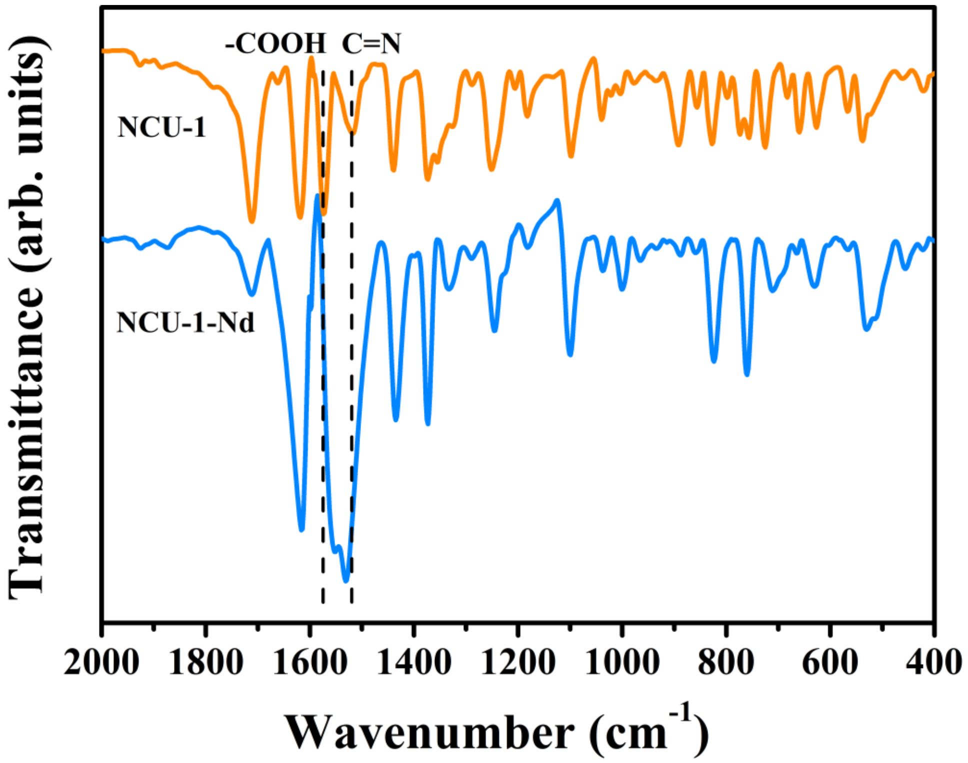

Supplementary Fig. 57 | FT-IR spectra of NCU-1 and NCU-1-Nd.

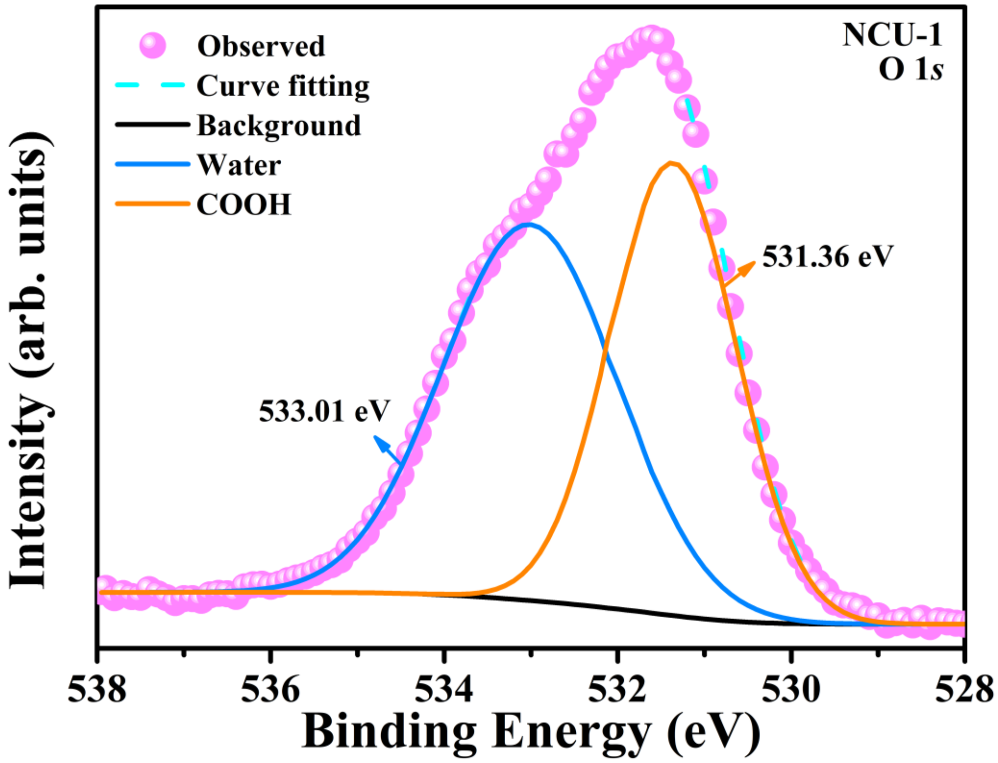

Supplementary Fig. 58 | The High-resolution O 1*s* of NCU-1.

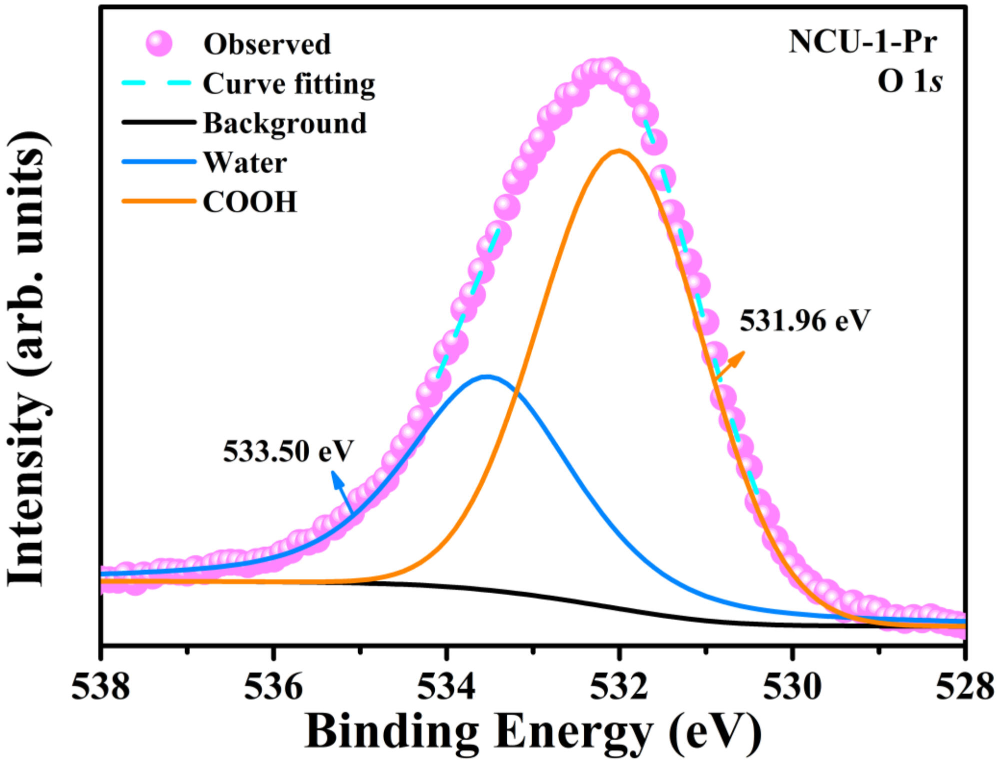

Supplementary Fig. 59 | The High-resolution O 1*s* of NCU-1-Pr.

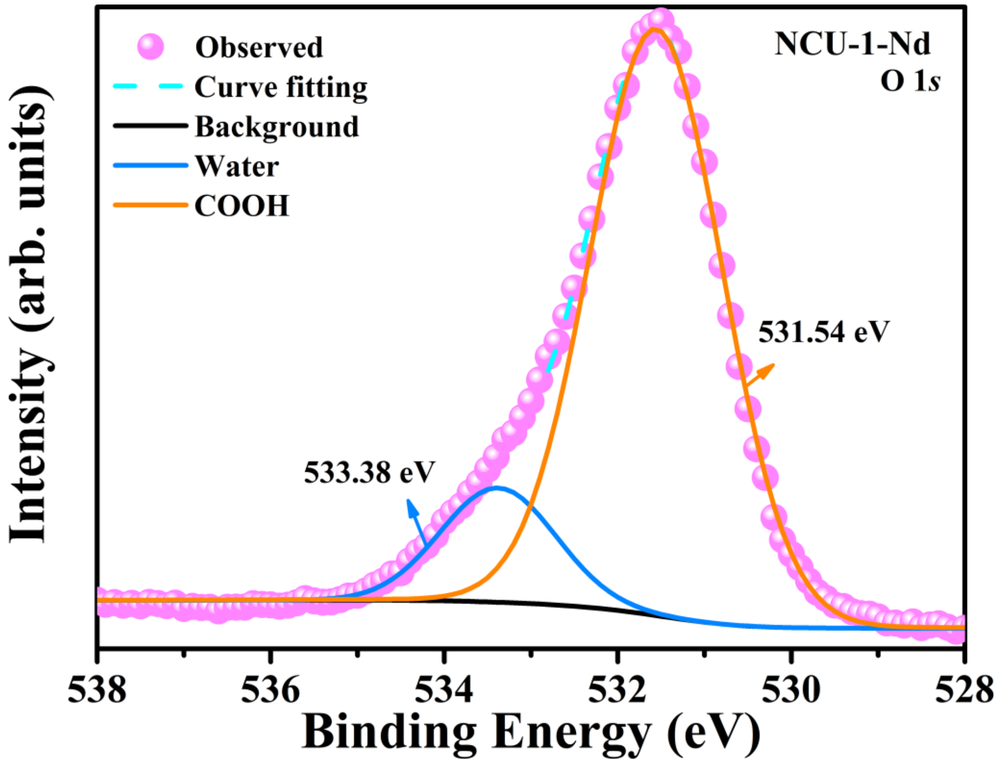

Supplementary Fig. 60 | The High-resolution O 1*s* of NCU-1-Nd.

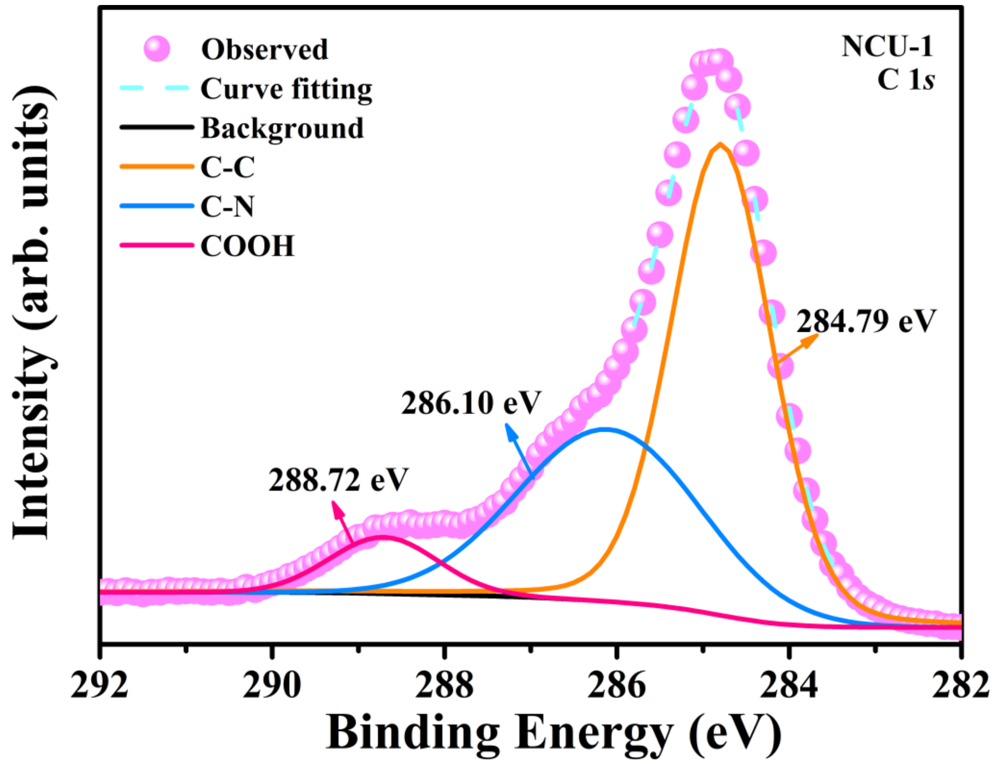

Supplementary Fig. 61 | The C 1*s* region of NCU-1.

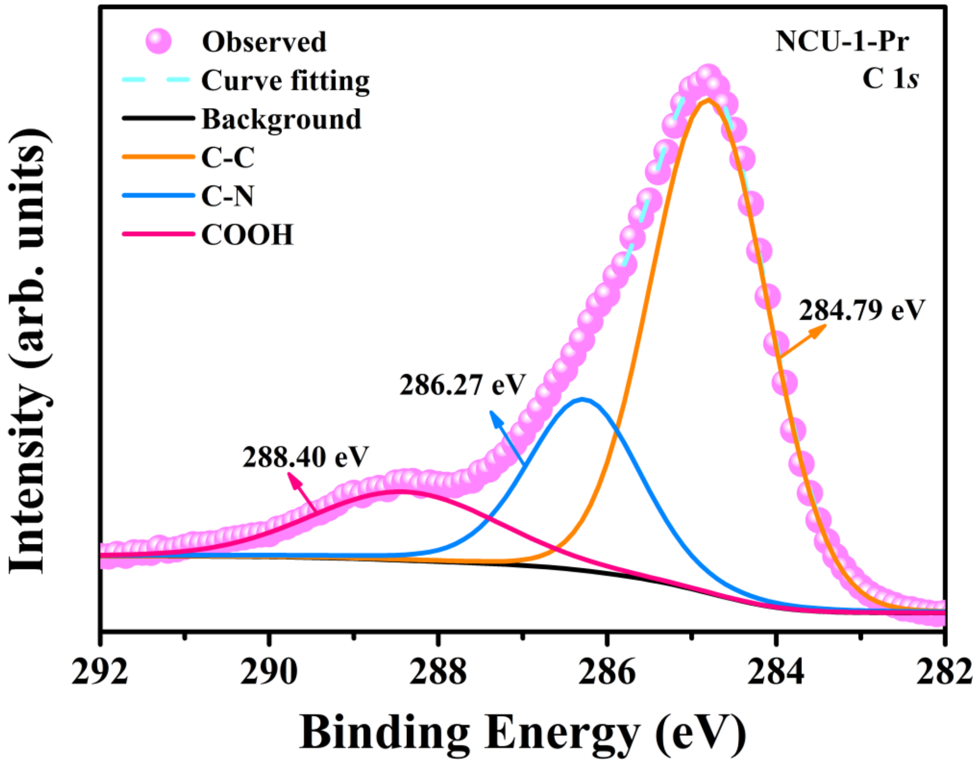

Supplementary Fig. 62 | The C 1*s* region of NCU-1-Pr.

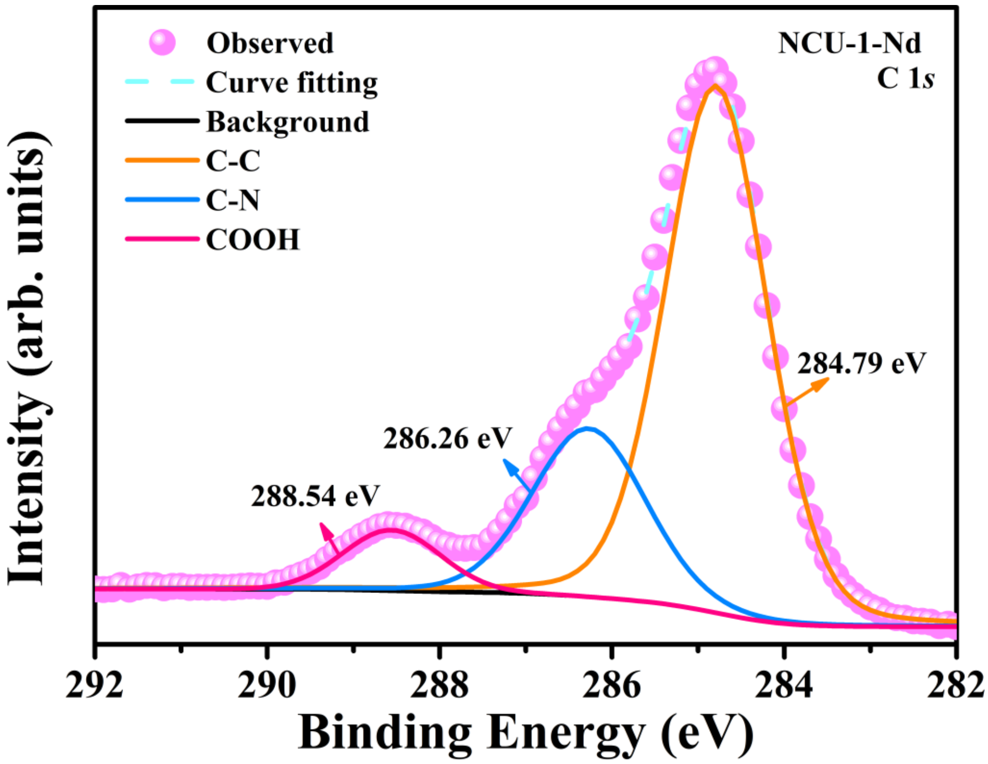

Supplementary Fig. 63 | The C 1*s* region of NCU-1-Nd.

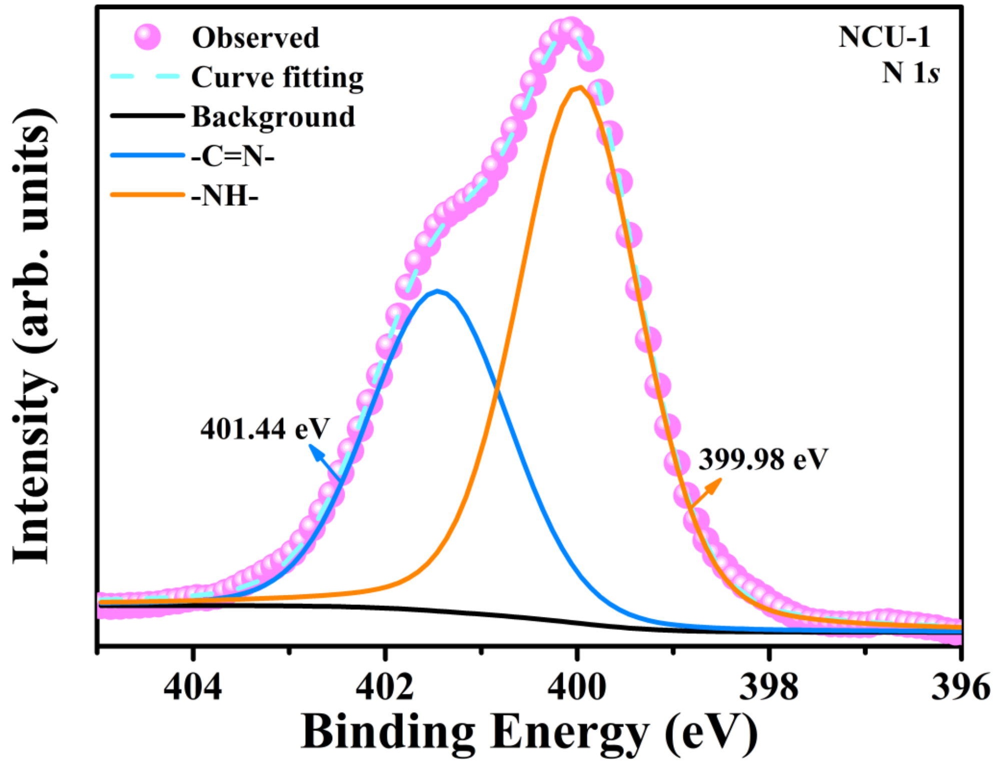

Supplementary Fig. 64 | The XPS spectra of the N 1*s* region of NCU-1.

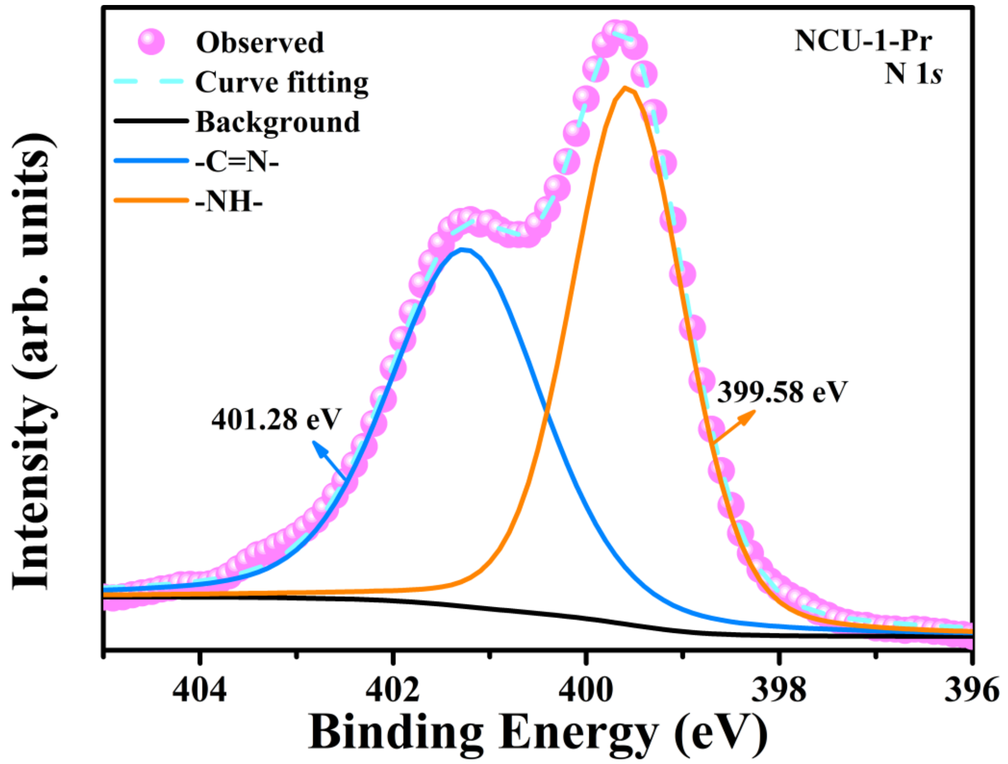

Supplementary Fig. 65 | The XPS spectra of the N 1*s* region of NCU-1-Pr.

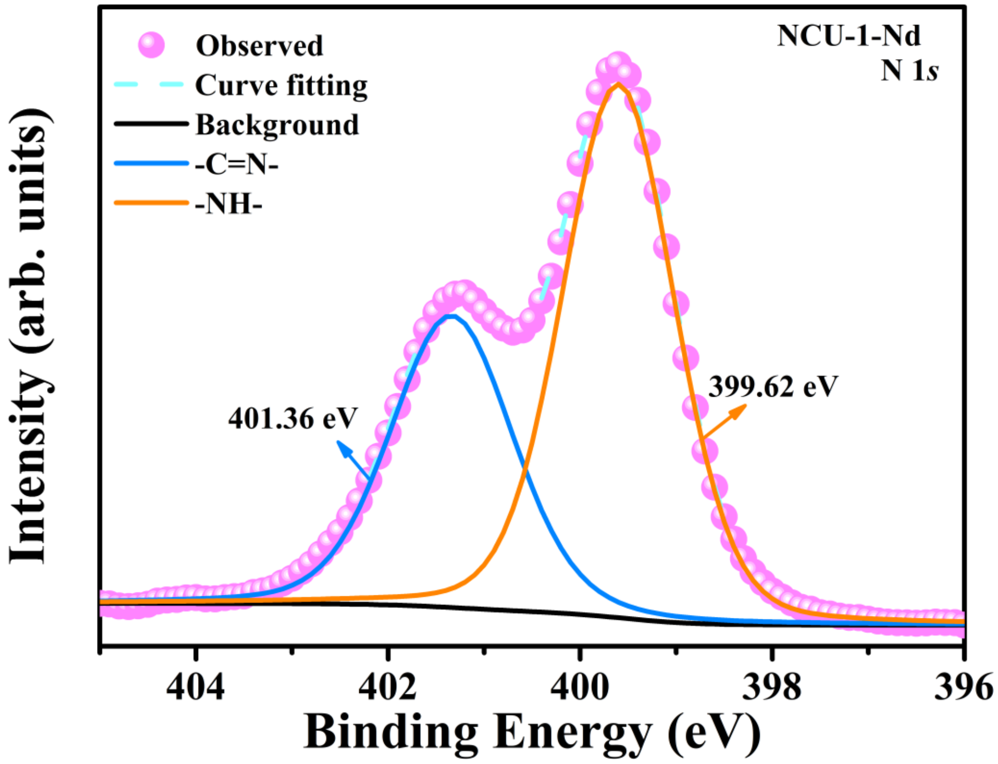

Supplementary Fig. 66 | The XPS spectra of the N 1*s* region of NCU-1-Nd.

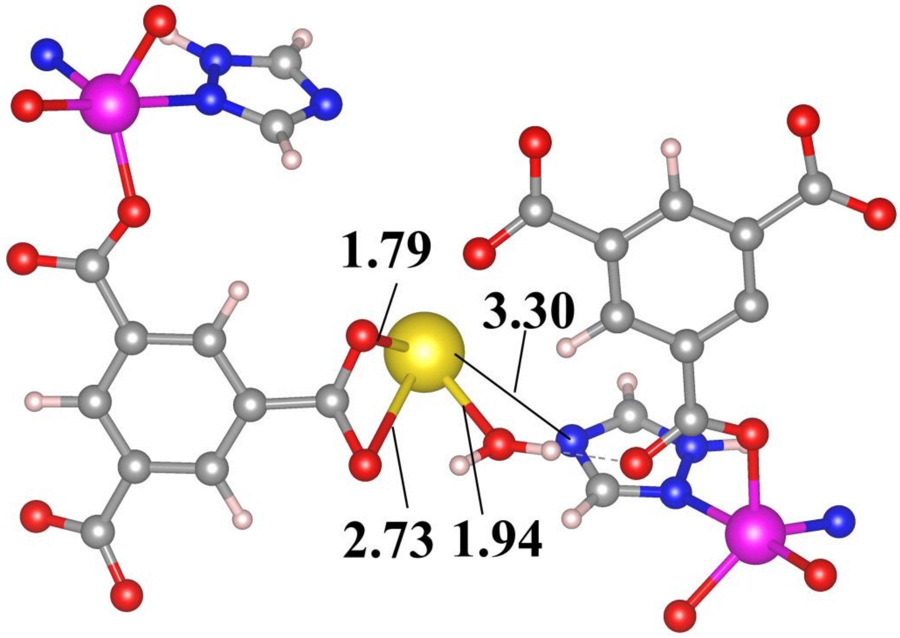

Supplementary Fig. 67 | The calculated adsorption binding sites of Pr^{3+}. (The labeled distance is measured in Å)

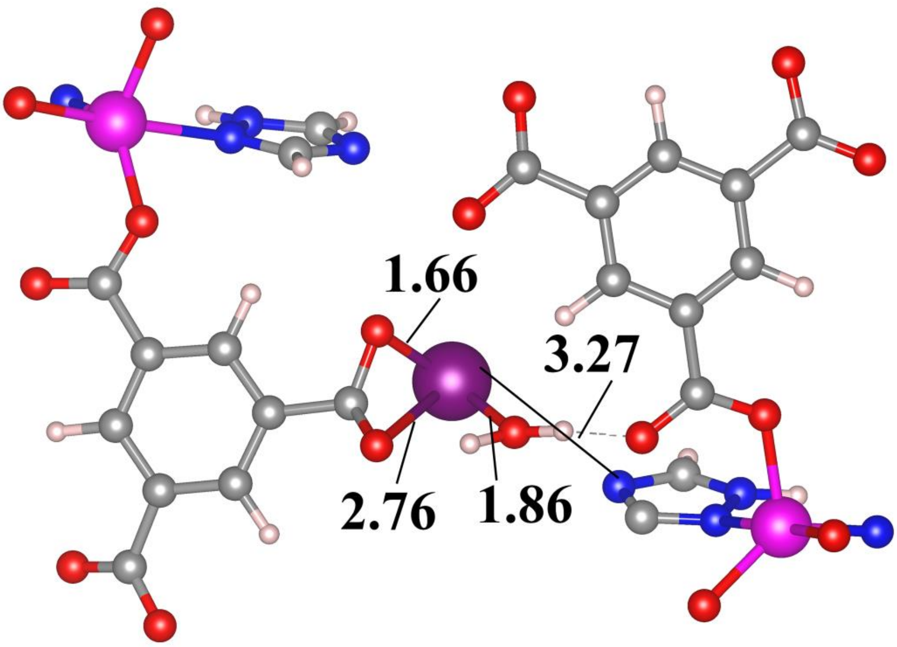

Supplementary Fig. 68 | The calculated adsorption binding sites of Nd^{3+}. (The labeled distance is measured in Å)

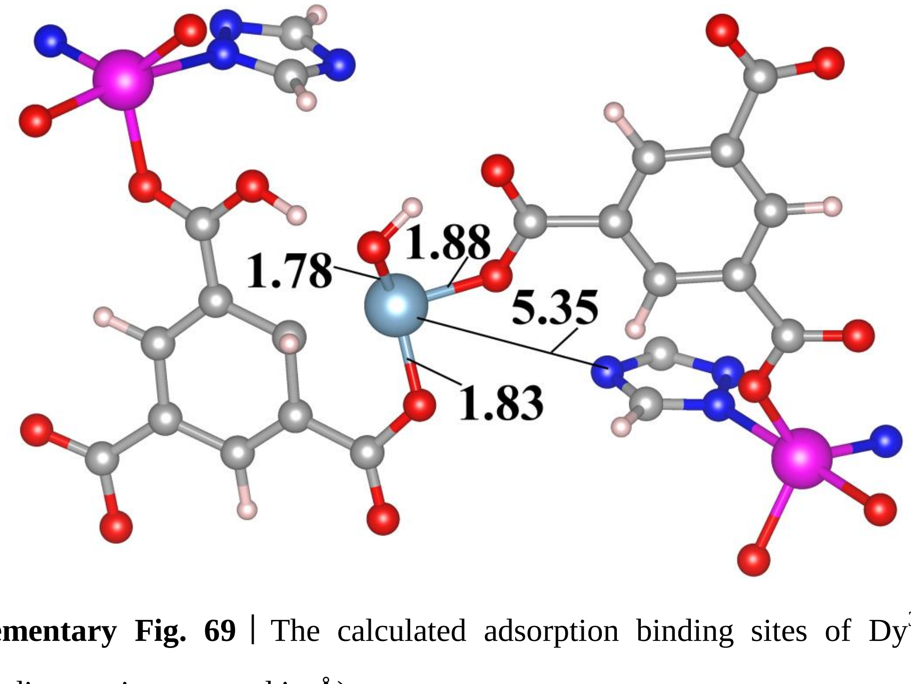

Supplementary Fig. 69 | The calculated adsorption binding sites of Dy^{3+}. (The labeled distance is measured in Å)

Supplementary Table 1 | Single-crystal X-ray structure refinement of NCU-1.

	NCU-1
CCDC No.	2141439
Empirical formula	$C_{25}H_{20}N_6O_8Zn$
Temperature/K	293(2)
Formula weight	599.885
Crystal system	orthorhombic
Space group	$Pna2_1$
a(Å)	9.74012(5)
b(Å)	17.02733(8)
c(Å)	14.43180(7)
α(°)	90
β(°)	90
γ(°)	90
Volume(Å^3)	2393.49(2)
Z	4
ρcalcd (g cm^{-3})	1.659
μ(mm^{-1})	2.009
F(000)	1224
R_{int}	0.0220
Data collected	42180
Independent data	4816
Goodness-of-fit	1.106
R_1[a] (I>2σ(I))	0.0220
wR_2[b] (I>2σ(I))	0.0583

Supplementary Table 2 | Elemental composition of mineral leachates and NCU-1 capture efficiency of rare earth in tailings collected from Ganzhou city.

REE (mg/L)				Interfering ions (mg/L)			
Metal ions	C_0 (mg/L)	C_e (mg/L)	Capture (%)	Metal ions	C_0 (mg/L)	C_e (mg/L)	Capture (%)
La	0.25	0	100	Al^{3+}	8.15	7.74	5.03
Ce^{3+}	0.13	0	100	Fe^{3+}	1.03	1.00	2.91
Pr^{3+}	0.35	0	100	Ca^{2+}	0.56	0.55	1.79
Nd^{3+}	0.81	0.01	98.77	Na^{+}	1.86	1.85	0.54
Sm^{3+}	0.46	0.01	97.83	Zn^{2+}	0.23	0.23	0
Eu^{3+}	0.32	0	100	Co^{2+}	0.01	0.01	0
Gd^{3+}	0.67	0.14	79.10	Cu^{2+}	0.01	0.01	0
Tb^{3+}	0.16	0.04	75.00	Ni^{2+}	0.02	0.02	0
Dy^{3+}	0.92	0.29	68.48	Pb^{2+}	0.06	0.06	0
Ho^{3+}	0.18	0.06	66.67				
Er^{3+}	0.53	0.2	62.26				
Tm^{3+}	0.09	0.03	66.67				
Yb^{3+}	0.44	0.19	56.82				
Lu^{3+}	0.37	0.19	48.65				

C_0, the initial concentration of metal ions in tailings collected from Ganzhou city. C_e, the equilibrium concentration of metal ions after adsorption by 10 mg NCU-1 at a rate of 120 rpm for 6 h.

Supplementary Table 3 | Fitting results of NCU-1 adsorption Pr^{3+}, Nd^{3+}, Eu^{3+}, Gd^{3+},Dy^{3+},Er^{3+}, and Lu^{3+} are based on the Langmuir and Freundlich models.

Sample	Langmuir models			Freundlich models		
	q_m (mg/g)	K_L (L/mg)	R^2	K_F	n	R^2
Pr^{3+}	446	0.112	0.988	100	3.18	0.957
Nd^{3+}	332	0.124	0.988	86.6	3.86	0.943
Eu^{3+}	226	0.150	0.994	54.8.0	3.47	0.959
Gd^{3+}	141	0.686	0.984	50.0	5.03	0.897
Dy^{3+}	134	0.061	0.956	23.7	3.22	0.876
Er^{3+}	98	0.018	0.997	25.7	3.76	0.774
Lu^{3+}	73	0.040	0.976	7.34	2.38	0.896

Supplementary Table 4 | Fitting results of NCU-1 adsorption Pr^{3+}, Nd^{3+}, Eu^{3+} , Gd^{3+}, Dy^{3+}, Er^{3+}, and Lu^{3+} are based on the Pseudo-first-order kinetic and Pseudo-second-order kinetic models.

Sample	Pseudo-first-order		Pseudo-second-order	
	k_1 (min^{-1})	R^2	k_2 (g (mg min)$^{-1}$)	R^2
Pr^{3+}	0.042	0.867	0.783	0.999
Nd^{3+}	0.033	0.791	1.802	0.996
Eu^{3+}	0.058	0.8896	0.254	0.999
Gd^{3+}	0.047	0.439	0.177	0.999
Dy^{3+}	0.087	0.893	0.195	0.999
Er^{3+}	0.075	0.491	0.511	0.999
Lu^{3+}	0.055	0.756	1.252	0.999

Supplementary Table 5 | K_d values of REE were captured by NCU-1 at various pH values from 2.0 to 6.0.

K_d^{Ln} (mL/g)	pH 2.0	pH 2.5	pH 3.0	pH 3.5	pH 4.0	pH 4.5	pH 5.0	pH 6.0
La	1.24×10^2	6.99×10^2	1.30×10^4	5.14×10^4	1.15×10^5	1.05×10^6	8.27×10^3	2.06×10^3
Ce	1.33×10^1	5.74×10^2	1.02×10^4	4.87×10^4	8.93×10^4	9.92×10^5	4.09×10^3	2.30×10^3
Pr	7.99	4.11×10^2	8.70×10^3	3.78×10^4	7.66×10^4	5.04×10^5	3.78×10^3	2.38×10^3
Nd	9.53×10^1	3.85×10^2	7.29×10^3	2.16×10^4	4.01×10^4	1.30×10^5	2.26×10^3	1.72×10^3
Sm	1.15×10^1	9.55×10^2	3.85×10^3	1.08×10^4	3.54×10^4	7.60×10^4	1.89×10^3	1.43×10^3
Eu	2.07×10^1	4.48×10^2	2.18×10^3	7.43×10^3	9.80×10^3	4.84×10^4	1.79×10^3	1.64×10^3
Gd	3.21×10^1	3.36×10^2	9.43×10^2	3.07×10^3	6.43×10^3	1.63×10^4	1.22×10^3	1.02×10^3
Tb	2.22×10^1	3.11×10^2	4.38×10^2	3.14×10^3	3.13×10^3	3.55×10^3	1.04×10^3	8.26×10^2
Dy	4.80×10^1	1.13×10^2	2.12×10^3	4.12×10^3	1.17×10^3	9.12×10^2	8.94×10^2	5.88×10^2
Ho	7.23	1.67×10^2	4.97×10^2	1.32×10^3	1.44×10^3	8.93×10^2	8.34×10^2	6.94×10^2
Er	1.24×10^1	2.67×10^2	4.67×10^2	1.73×10^3	1.09×10^3	7.51×10^2	6.20×10^2	5.66×10^2
Tm	1.19×10^2	3.77×10^2	6.27×10^2	7.30×10^2	7.87×10^2	6.53×10^2	5.80×10^2	4.62×10^2
Lu	9.64	1.56×10^2	1.78×10^2	5.55×10^2	6.18×10^2	5.98×10^2	4.64×10^2	3.58×10^2

Supplementary References

1. Dolomanov, O. V. et al. OLEX2: a complete structure solution, refinement and analysis program. *J. Appl. Crystallogr*. **42**, 339-341 (2009).
2. Sheldrick, G. Crystal structure refinement with SHELXL. *Acta Crystallogr. Sect. C* **71**, 3-8 (2015).
3. Hu, Q. H. et al. Synthesis of imidazolium-based cationic organic polymer for highly efficient and selective removal of ReO_4^-/TcO_4^-. *Chem. Eng. J.* **419**, 129546 (2021).
4. Sun, H. et al. Highly selective recovery of lanthanides by using a layered vanadate with acid and radiation resistance. *Angew. Chem. Int. Ed.* **59**, 1878-1883 (2020).
5. Zhao, X. et al. Size-selective crystallization of homochiral camphorate metal–organic frameworks for lanthanide separation. *J. Am. Chem. Soc.* **136**, 12572-12575 (2014).
6. Kresse, G. et al. Efficient iterative schemes for ab initio total-energy calculations using a plane-wave basis set. *Phys. Rev. B* **54**, 11169-11186 (1996).
7. Perdew, J. P. et al. Generalized gradient approximation made simple. *Phys. Rev. Lett.* **77**, 3865-3868 (1996).
8. Blöchl, P. E. Projector augmented-wave method. *Phys. Rev. B* **50**, 17953-17979 (1994).
